# Supplementary material for: Serological and viral prevalence of Oropouche virus (OROV): A systematic review and meta-analysis from 2000–2024 including human, animal, and vector surveillance studies
Source: PLoS Negl Trop Dis. 2025 Dec 29;19(12):e0013340. doi: 10.1371/journal.pntd.0013340 (PMC12799185; doi:10.1371/journal.pntd.0013340)
Supplement: S1 Text — File A: PRISMA Checklist. File B: Search Strategy. File C: PROSPERO Protocol Registration. File D: Full Inclusion and Exclusion Criteria. File E: Risk of Bias Tool Breakdown. Table A: Bibliographic summary of human studies. Table B: Bibliographic summary of vector studies. Table C: Risk of Bias breakdown for all studies. Table D: Summary of species in vector studies. Fig A. Sensitivity Analysis: Reported pooled seroprevalence from studies using screening assays only, sampling participants with febrile illness or suspected of Oropouche infection in 2000–2024. Fig B. Sensitivity Analysis: Reported pooled seroprevalence from studies using neutralizing assays, sampling participants with febrile illness or suspected of Oropouche infection in 2000–2024. Fig C. Sensitivity Analysis: Reported pooled seroprevalence from studies using screening assays only, sampling participants among asymptomatic general populations in 2000–2024. Fig D. Sensitivity Analysis: Reported pooled seroprevalence from studies using neutralizing assays, sampling participants among asymptomatic general populations in 2000–2024. (DOCX) [file pntd.0013340.s001.docx]

## Supplementary Materials

[Supplementary Materials 1](#_Toc217062349)

[File A: PRISMA Checklist 1](#_Toc217062350)

[File B: Search Strategy 5](#_Toc217062351)

[File C: PROSPERO Protocol Registration 8](#_Toc217062352)

[File D: Full inclusion and exclusion criteria 8](#_Toc217062353)

[File E: Risk of Bias Tool Breakdown 9](#_Toc217062354)

[Table A. Bibliographic summary of human studies. 12](#_Toc217062355)

[Table B. Bibliographic summary of vector studies. 17](#_Toc217062356)

[Table C. Risk of bias breakdown for all studies. 26](#_Toc217062357)

[Table D. Summary of species in vector studies 56](#_Toc217062358)

[Fig A. Sensitivity Analysis: Reported pooled seroprevalence from studies using screening assays only, sampling participants with febrile illness or suspected of Oropouche infection in 2000 to 2024. 57](#_Toc217062359)

[Fig B. Sensitivity Analysis: Reported pooled seroprevalence from studies using neutralizing assays, sampling participants with febrile illness or suspected of Oropouche infection in 2000 to 2024. 58](#_Toc217062360)

[Fig C. Sensitivity Analysis: Reported pooled seroprevalence from studies using screening assays only, sampling participants among asymptomatic general populations in 2000 to 2024. 59](#_Toc217062361)

[Fig D. Sensitivity Analysis: Reported pooled seroprevalence from studies using neutralizing assays, sampling participants among asymptomatic general populations in 2000 to 2024. 59](#_Toc217062362)

##

## File A: PRISMA Checklist

| **Section and Topic** | **Item #** | **Checklist item** | **Location where item is reported** |
| --- | --- | --- | --- |
| **TITLE** | | |  |
| Title | 1 | Identify the report as a systematic review. | In title, abstract, last paragraph of introduction. |
| **ABSTRACT** | | |  |
| Abstract | 2 | See the PRISMA 2020 for Abstracts checklist. | Page 1. |
| **INTRODUCTION** | | |  |
| Rationale | 3 | Describe the rationale for the review in the context of existing knowledge. | Introduction, paragraph 2. Page 3. |
| Objectives | 4 | Provide an explicit statement of the objective(s) or question(s) the review addresses. | Introduction, paragraph 3. Page 3-4. |
| **METHODS** | | |  |
| Eligibility criteria | 5 | Specify the inclusion and exclusion criteria for the review and how studies were grouped for the syntheses. | Methods, paragraph 2, page 4, and supplementary file D. |
| Information sources | 6 | Specify all databases, registers, websites, organisations, reference lists and other sources searched or consulted to identify studies. Specify the date when each source was last searched or consulted. | Methods, paragraph 1, page 4. |
| Search strategy | 7 | Present the full search strategies for all databases, registers and websites, including any filters and limits used. | Supplementary file B. |
| Selection process | 8 | Specify the methods used to decide whether a study met the inclusion criteria of the review, including how many reviewers screened each record and each report retrieved, whether they worked independently, and if applicable, details of automation tools used in the process. | Methods, paragraph 2, page 4. |
| Data collection process | 9 | Specify the methods used to collect data from reports, including how many reviewers collected data from each report, whether they worked independently, any processes for obtaining or confirming data from study investigators, and if applicable, details of automation tools used in the process. | Methods, paragraph 3, page 4. |
| Data items | 10a | List and define all outcomes for which data were sought. Specify whether all results that were compatible with each outcome domain in each study were sought (e.g. for all measures, time points, analyses), and if not, the methods used to decide which results to collect. | Methods, paragraph 3-5, page 4-5. Also available in the data dictionary, which is in our data availability statement and open-access database at [serotracker.com](http://serotracker.com) , ArboTracker dashboard, also cited on the top of page 5. |
|  | 10b | List and define all other variables for which data were sought (e.g. participant and intervention characteristics, funding sources). Describe any assumptions made about any missing or unclear information. | Methods, paragraph 3-5, page 4-5. Also available in the data dictionary and protocol, which are in our data availability statement and open-access database at [serotracker.com](http://serotracker.com) , ArboTracker dashboard. |
| Study risk of bias assessment | 11 | Specify the methods used to assess risk of bias in the included studies, including details of the tool(s) used, how many reviewers assessed each study and whether they worked independently, and if applicable, details of automation tools used in the process. | Methods paragraph 4, page 5, and supplementary file E. |
| Effect measures | 12 | Specify for each outcome the effect measure(s) (e.g. risk ratio, mean difference) used in the synthesis or presentation of results. | Methods paragraph 5, page 5. |
| Synthesis methods | 13a | Describe the processes used to decide which studies were eligible for each synthesis (e.g. tabulating the study intervention characteristics and comparing against the planned groups for each synthesis (item #5)). | Methods paragraph 5, page 5. |
|  | 13b | Describe any methods required to prepare the data for presentation or synthesis, such as handling of missing summary statistics, or data conversions. | Methods, paragraph 3-5, page 4-5. |
|  | 13c | Describe any methods used to tabulate or visually display results of individual studies and syntheses. | Methods paragraph 5, page 5. |
|  | 13d | Describe any methods used to synthesize results and provide a rationale for the choice(s). If meta-analysis was performed, describe the model(s), method(s) to identify the presence and extent of statistical heterogeneity, and software package(s) used. | Methods paragraph 5, page 5. |
|  | 13e | Describe any methods used to explore possible causes of heterogeneity among study results (e.g. subgroup analysis, meta-regression). | Methods paragraph 5, page 5. |
|  | 13f | Describe any sensitivity analyses conducted to assess robustness of the synthesized results. | N/A |
| Reporting bias assessment | 14 | Describe any methods used to assess risk of bias due to missing results in a synthesis (arising from reporting biases). | N/A |
| Certainty assessment | 15 | Describe any methods used to assess certainty (or confidence) in the body of evidence for an outcome. | Methods paragraph 5, page 5. |
| **RESULTS** | | |  |
| Study selection | 16a | Describe the results of the search and selection process, from the number of records identified in the search to the number of studies included in the review, ideally using a flow diagram. | Results paragraph 1, page 5. Figure 1, PRISMA flowchart. |
|  | 16b | Cite studies that might appear to meet the inclusion criteria, but which were excluded, and explain why they were excluded. | N/A |
| Study characteristics | 17 | Cite each included study and present its characteristics. | Page 6. |
| Risk of bias in studies | 18 | Present assessments of risk of bias for each included study. | Last paragraph of results on page 11, and supplementary file E and table C. |
| Results of individual studies | 19 | For all outcomes, present, for each study: (a) summary statistics for each group (where appropriate) and (b) an effect estimate and its precision (e.g. confidence/credible interval), ideally using structured tables or plots. | Supplementary Table A and B |
| Results of syntheses | 20a | For each synthesis, briefly summarise the characteristics and risk of bias among contributing studies. | Supplementary Table C |
|  | 20b | Present results of all statistical syntheses conducted. If meta-analysis was done, present for each the summary estimate and its precision (e.g. confidence/credible interval) and measures of statistical heterogeneity. If comparing groups, describe the direction of the effect. | Figure 2-4, Results paragraph 4-6 |
|  | 20c | Present results of all investigations of possible causes of heterogeneity among study results. | Discussion paragraph 6 |
|  | 20d | Present results of all sensitivity analyses conducted to assess the robustness of the synthesized results. | N/A |
| Reporting biases | 21 | Present assessments of risk of bias due to missing results (arising from reporting biases) for each synthesis assessed. | N/A |
| Certainty of evidence | 22 | Present assessments of certainty (or confidence) in the body of evidence for each outcome assessed. | Figure 2-4, Results paragraph 4-6 |
| **DISCUSSION** | | |  |
| Discussion | 23a | Provide a general interpretation of the results in the context of other evidence. | Human results in Paragraphs 1-4 of discussion, vector/animal results summarized in paragraphs 5-6. Page 11-13. |
|  | 23b | Discuss any limitations of the evidence included in the review. | Discussion paragraph 7. Page 13. |
|  | 23c | Discuss any limitations of the review processes used. | Discussion paragraph 7. Page 13. |
|  | 23d | Discuss implications of the results for practice, policy, and future research. | Page 14. |
| **OTHER INFORMATION** | | |  |
| Registration and protocol | 24a | Provide registration information for the review, including register name and registration number, or state that the review was not registered. | Abstract and methods paragraph 1. |
|  | 24b | Indicate where the review protocol can be accessed, or state that a protocol was not prepared. | Methods paragraph 1 (PROSPERO) and also available at the ArboTracker online dashboard. |
|  | 24c | Describe and explain any amendments to information provided at registration or in the protocol. | N/A |
| Support | 25 | Describe sources of financial or non-financial support for the review, and the role of the funders or sponsors in the review. | Conflict disclosure statement page 15 |
| Competing interests | 26 | Declare any competing interests of review authors. | Data availability statement page 15-16 |
| Availability of data, code and other materials | 27 | Report which of the following are publicly available and where they can be found: template data collection forms; data extracted from included studies; data used for all analyses; analytic code; any other materials used in the review. | Data availability statement page 15-16 |

*From:*  Page MJ, McKenzie JE, Bossuyt PM, Boutron I, Hoffmann TC, Mulrow CD, et al. The PRISMA 2020 statement: an updated guideline for reporting systematic reviews. BMJ 2021;372:n71. doi: 10.1136/bmj.n71. This work is licensed under CC BY 4.0. To view a copy of this license, visit<https://creativecommons.org/licenses/by/4.0/>

## File B: Search Strategy

Database: EMBASE

Dates: start date not defined to September 12, 2024

| **#** | **Search terms** |
| --- | --- |
| 1 | exp Oropouche virus/ OR exp Oropouche orthobunyavirus/ OR Oropouche.mp. OR OROV.mp. OR Iquitos virus.mp. OR IQTV.mp. OR Madre de Dios virus.mp. OR MDDV.mp. OR Perdoes virus.mp. OR PDEV.mp. |
| 2 | study.mp. OR studies.mp. OR survey*.mp. OR seroprevalence/ OR serosurvey*.mp. OR sero-survey*.mp. OR serosurveillance.mp. OR exp monitoring/ OR surveillance.mp. |
| 3 | exp antibody/ OR antibod*.mp. OR virus.mp. OR exp virus/ OR viral.mp. OR exp DNA/ OR DNA.mp. OR exp antigen/ OR antigen*.mp. OR seroprevalence.mp. OR exp seroprevalence/ OR sero-prevalence.mp. OR prevalence.mp. OR exp prevalence/ OR incidence.mp. OR exp incidence/ |
| 4 | detect*.mp. OR test*.mp. OR assay*.mp. OR immunoassay*.mp. OR exp immunoassay/ OR PCR.mp. OR exp polymerase chain reaction/ |
| 5 | 1 AND 2 AND 3 AND 4 |

Database: Europe PMC

Dates: January 1, 1900 to September 12, 2024

Notes: Pre-prints only

| **#** | **Search terms** |
| --- | --- |
| 1 | Oropouche OR "ORO virus" OR oropuche OR ORVO OR "Madre de Dios virus" OR MDDV OR "Perdoes virus" OR PDEV OR "Iquitos virus" OR IQTV |
| 2 | study OR studies OR survey* OR serosurvey* OR sero-survey* OR surveillance |
| 3 | antibod* OR virus OR viral OR DNA OR antigen OR seroprevalence OR sero-prevalence OR prevalence OR incidence |
| 4 | detect* OR test* OR assay* OR immunoassay* OR PCR |
| 5 | 1 AND 2 AND 3 AND 4 |
| 6 | AND (FIRST_PDATE:[1900-01-01 TO 2024-09-12]) |
| 7 | AND (SRC:PPR) |

Database: LILACS

Dates: start date not defined to September 12, 2024

| **#** | **Search terms** |
| --- | --- |
| 1 | oropouche OR OROV OR "Iquitos virus" OR IQTV OR "Madre de Dios virus" OR MDDV OR "Perdoes virus" OR PDEV |
| 2 | study OR studies OR survey* OR serosurvey OR sero-survey* OR surveillance OR sero-surveillance OR serosurveillance |
| 3 | detect* OR test* OR assay* OR immunoassay* OR PCR |
| 4 | antibod* OR virus OR viral OR DNA OR antigen* OR seroprevalence OR sero-prevalence OR prevalence OR incidence |
| 5 | 1 AND 2 AND 3 AND 4 |

Database: Medline

Dates: start date not defined to September 12, 2025

| **#** | **Search terms** |
| --- | --- |
| 1 | exp Oropouche virus/ OR exp Oropouche orthobunyavirus/ OR Oropouche.mp. OR OROV.mp. |
| 2 | study.mp. OR studies.mp. OR survey*.mp. OR serosurvey*.mp. OR sero-survey*.mp. OR serosurveillance.mp. OR exp monitoring/ OR surveillance.mp. |
| 3 | exp antibody/ OR antibod*.mp. OR virus.mp. OR exp virus/ OR viral.mp. OR exp DNA/ OR DNA.mp. OR exp antigen/ OR antigen*.mp. OR seroprevalence.mp. OR exp seroprevalence/ OR sero-prevalence.mp. OR prevalence.mp. OR exp prevalence/ OR incidence.mp. OR exp incidence/ |
| 4 | detect*.mp. OR test*.mp. OR assay*.mp. OR immunoassay*.mp. OR exp immunoassay/ OR PCR.mp. OR exp polymerase chain reaction/ |
| 5 | 1 AND 2 AND 3 AND 4 |

Database: Pubmed

Dates: start date not defined to September 12, 2024

| **#** | **Search terms** |
| --- | --- |
| 1 | "oropouche"[All Fields] OR "OROV"[All Fields] OR "Iquitos virus" [All Fields] OR "IQTV" [All Fields] OR "Madre de Dios virus" [All Fields] OR "MDDV" [All Fields] OR "Perdoes virus" [All Fields] OR "PDEV" [All Fields] |
| 2 | "studies"[All Fields] OR "study"[All Fields] OR "study s"[All Fields] OR "studying"[All Fields] OR "studys"[All Fields] OR "studies"[All Fields] OR "study"[All Fields] OR "study s"[All Fields] OR "studying"[All Fields] OR "studys"[All Fields] OR "survey*"[All Fields] OR "serosurvey*"[All Fields] OR "sero survey*"[All Fields] OR "serosurveillance"[All Fields] OR "epidemiology"[MeSH Subheading] OR "epidemiology"[All Fields] OR "surveillance"[All Fields] OR "epidemiology"[MeSH Terms] OR "surveilance"[All Fields] OR "surveillances"[All Fields] OR "surveilled"[All Fields] OR "surveillence"[All Fields] |
| 3 | "antibod*"[All Fields] OR ("virology"[MeSH Subheading] OR "virology"[All Fields] OR "viruses"[All Fields] OR "viruses"[MeSH Terms] OR "virus s"[All Fields] OR "viruse"[All Fields] OR "virus"[All Fields]) OR ("virally"[All Fields] OR "virals"[All Fields] OR "virology"[MeSH Terms] OR "virology"[All Fields] OR "viral"[All Fields]) OR ("dna"[MeSH Terms] OR "dna"[All Fields]) OR "antigen*"[All Fields] OR ("seroepidemiologic studies"[MeSH Terms] OR ("seroepidemiologic"[All Fields] AND "studies"[All Fields]) OR "seroepidemiologic studies"[All Fields] OR "seroprevalence"[All Fields] OR "seroprevalences"[All Fields] OR "seroprevalance"[All Fields] OR "seroprevalances"[All Fields] OR "seroprevalency"[All Fields] OR "seroprevalent"[All Fields]) OR "sero-prevalence"[All Fields] OR ("epidemiology"[MeSH Subheading] OR "epidemiology"[All Fields] OR "prevalence"[All Fields] OR "prevalence"[MeSH Terms] OR "prevalance"[All Fields] OR "prevalences"[All Fields] OR "prevalence s"[All Fields] OR "prevalent"[All Fields] OR "prevalently"[All Fields] OR "prevalents"[All Fields]) OR ("epidemiology"[MeSH Subheading] OR "epidemiology"[All Fields] OR "incidence"[All Fields] OR "incidence"[MeSH Terms] OR "incidences"[All Fields] OR "incident"[All Fields] OR "incidents"[All Fields]) |
| 4 | "detect*"[All Fields] OR "test*"[All Fields] OR "assay*"[All Fields] OR "immunoassay*"[All Fields] OR "PCR"[All Fields] |
| 5 | 1 AND 2 AND 3 AND 4 |

Database: Web of Science

Dates: start date not defined to September 12, 2024

| **#** | **Search terms** |
| --- | --- |
| 1 | ALL=(oropouche OR ORVO OR "Madre de Dios virus" OR MDDV OR "Perdoes virus" OR PDEV OR "Iquitos virus" OR IQTV) |
| 2 | ALL = (study OR studies OR survey* OR serosurvey* OR sero-survey* OR surveillance) |
| 3 | ALL=(antibod* OR virus OR viral OR DNA OR antigen OR seroprevalence OR sero-prevalence OR prevalence OR incidence) |
| 4 | ALL=(detect* OR test* OR assay* OR immunoassay* OR PCR) |
| 5 | 1 AND 2 AND 3 AND 4 |

## File C: PROSPERO Protocol Registration

The following protocol is pulled from our most recent PROSPERO protocol registration (March 17, 2025 version) CRD42024551000.

<https://www.crd.york.ac.uk/PROSPERO/view/CRD42024551000>

## File D: Full inclusion and exclusion criteria

Criteria for including evidence (must meet all the criteria to be included)

| **Characteristics** | **Criteria for inclusion** |
| --- | --- |
| Population | - Humans of any age - Animal populations - Insect populations - Including studies that only included symptomatic individuals or those with suspected OROV |
| Study design | - Sero-surveys – defined as the collection and testing of serum (or proxy such as oral fluid) specimens to estimate the prevalence of antibodies or T-cells against OROV as an indicator of immunity, and/or - Molecular or viral epidemiology studies to estimate the positivity rate of direct virus detection (through any of the following: PCR, sequencing, immunofluorescence, antigen) for OROV - Samples from a defined population over a specified period of time, including symptomatic persons and those with suspected disease - Cross-sectional, repeated cross-sectional, evaluations of serological tests, case-control, and cohort study designs, with serology or PCR measurements at single time points or repeated at multiple time points |
| *Special design | - Include systematic reviews and meta-analyses of seroprevalence studies for the purpose of tracking evidence-synthesis efforts |
| Sampling | - Any sampling method |
| Types of evidence | - Published or preprinted academic literature, - Grey literature (government, institutional, or meeting reports) - Media reports - Slide deck presentations were included if we could identify the person giving the presentation and the date of the presentation, and the institution |
| Outcome measures | - Reports a seroprevalence estimate (proportion of the population with detectable antibodies; including negative results i.e. undetectable antibodies), or - Reports a prevalence estimate (proportion of the population with detectable viral nucleic acids; including negative results i.e. undetectable nucleic acids) - Reports the number of participants enrolled in the study (denominator) - Reports study sampling period (date or week, can be inferred from month/year) - Reports the locations at which the study took places such that they could be categorized as neighbourhood, city, state/province/territory, or country |
| Languages | - Any |

Criteria for excluding evidence (if any met then exclude)

| **Characteristics** | **Criteria for exclusion** |
| --- | --- |
| Population | - Laboratory/experimental (non-field study population: e.g., *in silico*, *in vitro;* non-natural infection) |
| Study design | - Study designs other than cross-sectional or cohort design (such as case reports, study protocols) |
| Sampling | - N/A |
| Types of evidence | - Multimedia sources of data (audio clips, video clips) were excluded due to the feasibility of extracting. Slide deck presentations were excluded if we could not identify the person giving the presentation and the date of the presentation |
| Outcome measures | - Does not report OROV prevalence or sufficient information to calculate a prevalence of OROV - Does not report study sampling end date/week, or cannot be reasonably inferred with the available information - Does not report the number of participants included in the study (sample denominator) - Does not report the location at which the study took place |
| Language | - N/A |

## File E: Risk of Bias Tool Breakdown

To assess risk of bias, a decision rule assigned a rating of low, moderate, or high risk of bias to each study based on the specific combination of JBI checklist ratings for that study [[1]](https://www.zotero.org/google-docs/?e6PLHh). This decision rule was developed based on guidance on estimating disease prevalence [[17,18]](https://www.zotero.org/google-docs/?Oc2SJT) and was validated against assessments derived manually by two independent reviewers for 2,070 seroprevalence studies in the SeroTracker database, showing good agreement with manual review (intraclass correlation 0.77, 95% CI 0.74-0.80) in a recent paper [[72]](https://www.zotero.org/google-docs/?HgfqVa).

​

| **Item 1: Was the sample frame appropriate to address the target population?** | |
| --- | --- |
| Yes | Sample frame described and approximated the target population |
| No | Sample frame did not approximate the target population (e.g., blood donors do not represent general population, doctors do not represent all health care providers) |
| Exclude | Sample frame not described |
| *Notes | The term “target population” should not be taken to infer every individual from everywhere or with similar disease or exposure characteristics. Instead, give consideration to specific population characteristics in the study, including age range, gender, morbidities, medications, and other potentially influential factors. For example, a sample frame may not be appropriate to address the target population if a certain group has been used (such as those working for one organisation, or one profession) and the results then inferred to the target population (i.e. working adults). A sample frame may be appropriate when it includes almost all the members of the target population (i.e. a census, or a complete list of participants or complete registry data). |

| **Item 2: Were study participants recruited in an appropriate way?** | |
| --- | --- |
| Yes | Convenience sampling, probability sampling method (simple or stratified random) or entire sample (e.g., an entire town) was used |
| No | Sampling method not reported |
| Exclude |  |

| **Item 3: Was the sample size adequate?** | |
| --- | --- |
| Yes | >99 |
| No | <99 |
| Exclude | Sample size not reported |
| *Notes |  |

| **Item 4: Were the study subjects and setting described in detail?** | |
| --- | --- |
| Yes | Average age and distribution of gender/sex provided |
| No | Neither age or gender/sex is provided, or only one of age and gender/sex is provided |

| **Item 5: Was data analysis conducted with sufficient coverage of the identified sample?** | |
| --- | --- |
| Yes | The demographic characteristics (gender/sex, age, and ethnicity) of the sample are at least somewhat representative of the population in both the main and sub-group analyses |
| No | The demographic characteristics (gender/sex, age, and ethnicity) of the sample are not representative of the population in both the main and sub-group analyses |
| Unclear | Information is not provided about demographic characteristics of the sample (gender/sex, age, and ethnicity) |

| **Item 6: Were valid methods used for the identification of the condition?** | |
| --- | --- |
| Yes | Serology or viral test type was reported |
| No | Serology or viral test type was not reported |
| Exclude |  |

| **Item 7: Was the condition measured in a standard, reliable way for all participants?** | |
| --- | --- |
| Yes | The same serology test was used for all participants |
| No | Different serology tests were used for participants |
| Unclear | No details were provided about which participants received which serology tests |

| **Item 8: Was there appropriate statistical analysis?** | |
| --- | --- |
| Yes | Corrects for population characteristics OR the sample is somewhat representative of the population, and provides the information necessary to determine the numerator, denominator, prevalence estimate, and confidence interval. |
| No | Does not correct for population characteristics and the sample is not likely representative of the population or does not provide the information necessary to determine the numerator, denominator, prevalence estimate, and confidence interval. |

| **Item 9: Overall risk of bias** | |
| --- | --- |
| Low | The estimates are very likely correct for the target population. To obtain a low risk of bias classification, all criteria must be met or departures from the criteria must be minimal and unlikely to impact on the validity and reliability of the prevalence estimate. These include sample sizes that are just below the threshold when all other criteria are met, reporting only some of characteristics of the sample, test characteristics below the threshold but corrections for the test performance, and response rates that are just below the threshold in the context of probability based sampling of an appropriate sampling frame with population weighted seroprevalence estimates. |
| Moderate | The estimates are likely correct for the target population. To obtain a moderate risk of bias classification, most criteria must be met and departures from the criteria are likely to have only a small impact on the validity and reliability of the prevalence estimates. |
| High | The estimates are not likely correct for the target population. To obtain a high risk of bias, many criteria must not be met or departures from criteria are likely to have a major impact on the validity and reliability of the prevalence estimates. |
| Unclear | There was insufficient information to assess the risk of bias. |

##

## Table A. Bibliographic summary of human studies.

| Author | Sampling year | Country | State or province | Population | Estimate type | Positive cases | Number tested | Prevalence (%) | Assay type | Assay target | Data extractor | Date extracted |
| --- | --- | --- | --- | --- | --- | --- | --- | --- | --- | --- | --- | --- |
| F. P. Pinheiro[1] | 1975 | Brazil | Para | Students and Daycares | Seroprevalence | 45 | 112 | 0.402 | HAI | NAb | Emilie Toews | 10/16/2024 |
| James LeDuc[2] | 1978 | Brazil | Para | Febrile patients | Seroprevalence | 164 | 555 | 0.295 | HAI | NR | Mairead Whelan | 10/16/2024 |
| Ronaldo B. Freitas[3] | 1979 | Brazil | Para | Febrile patients | Viral Prevalence | 57 | 546 | 0.104 | Viral isolation | NAb | Emilie Toews | 10/16/2024 |
| Ronaldo B. Freitas[3] | 1979 | Brazil | Para | Community | Seroprevalence | 381 | 2,975 | 0.128 | HAI | NAb | Emilie Toews | 10/16/2024 |
| Carlos Borborema[4] | 1980 | Brazil | Amazonas | Community | Seroprevalence | 110 | 1,018 | 0.108 | HAI | NAb | Emilie Toews | 10/16/2024 |
| Pedro Fernando da Costa Vasconcelos[5] | 1988 | Brazil | Maranhão | Febrile patients | Seroprevalence | 256 | 394 | 0.650 | Other | IgM,NAb | Shaila Akter | 10/16/2024 |
| Douglas Watts[6] | 1994 | Peru |  | Essential non-healthcare workers | Seroprevalence | 6 | 68 | 0.088 | ELISA | IgM | Emilie Toews | 10/16/2024 |
| Amélia Rosa[7] | 1994 | Brazil | Pará | Community | Seroprevalence | 490 | 592 | 0.828 | Other | IgM,NAb | Shaila Akter | 10/16/2024 |
| Kathy Baisley[8] | 1996 | Peru |  | Community | Seroprevalence | 828 | 2,454 | 0.337 | ELISA | IgG | Emilie Toews | 10/16/2024 |
| José Tavares-Neto[9] | 1999 | Brazil | Acre | Community | Seroprevalence | 20 | 380 | 0.053 | Other | NR | Emilie Toews | 10/16/2024 |
| Douglas M. Watts[10] | 1999 | Peru |  | Febrile patients | Seroprevalence | 203 | 19,798 | 0.010 | ELISA | IgG,IgM | Harriet Ware | 10/16/2024 |
| Regina Maria Pinto De Figueiredo[11] | 1999 | Brazil | Amazonas | Residual sera | Seroprevalence | 3 | 35 | 0.086 | ELISA | IgM | Mairead Whelan | 10/10/2024 |
| Raimunda do Socorro da Silva Azevedo[12] | 2004 | Brazil | Pará State | Positive (PCR) or suspected cases | Seroprevalence | 284 | 734 | 0.387 | ELISA | IgM | Sabah Shaikh | 10/16/2024 |
| Stephen Manock[13] | 2004 | Ecuador | Pastaza | Febrile patients | Seroprevalence | 2 | 608 | 0.003 | ELISA | IgM | Shaila Akter | 10/16/2024 |
| Marcio Nunes[14] | 2005 | Brazil | Para State | Other | Seroprevalence | 22 | 2,766 | 0.008 | HAI | NAb | Emilie Toews | 10/16/2024 |
| Helena Vasconcelos[15] | 2006 | Brazil | Pará | Febrile patients | Seroprevalence | 480 | 1,113 | 0.431 | HAI | NAb | Emilie Toews | 10/16/2024 |
| Ana Carolina Bernardes Terzian[16] | 2006 | Brazil | Acre | Febrile patients | Viral Prevalence | 1 | 69 | 0.014 | RT-PCR | S segment (OROV only) | Caseng Zhang | 10/16/2024 |
| Patricia Aguilar[17] | 2006 | Peru | Maynas | Febrile patients | Seroprevalence | 154 | 1,037 | 0.149 | ELISA | IgG | Shaila Akter | 10/16/2024 |
| Brett Forshey[18] | 2007 | Bolivia (Plurinational State of) | Ñuflo de Chaves | Febrile patients | Viral Prevalence | 593 | 42,354 | 0.014 | RT-PCR | NR | Shaila Akter | 10/16/2024 |
| Ana Cecilia Ribeiro Cruz[19] | 2008 | Brazil | Pará | Community | Seroprevalence | 113 | 3,194 | 0.035 | HAI | NAb | Emilie Toews | 10/16/2024 |
| Maria Paula Mourão[20] | 2008 | Brazil | Amazonas | Febrile patients | Seroprevalence | 128 | 631 | 0.203 | ELISA | IgM | Caseng Zhang | 10/16/2024 |
| Pedro P. Alvarez[21] | 2010 | Peru | San Martin | Febrile patients | Seroprevalence | 108 | 675 | 0.160 | ELISA | IgM | Emilie Toews | 10/16/2024 |
| Valquiria do Carmo Alves Martins[22] | 2011 | Brazil | Amazonas | Positive cases of a different arbovirus | Viral Prevalence | 0 | 677 | 0.000 | RT-PCR | NR | Emilie Toews | 10/16/2024 |
| Sara Castro[23] | 2011 | Peru | Cajamarca | Positive (PCR) or suspected cases | Seroprevalence | 17 | 26 | 0.654 | ELISA | IgM | Emilie Toews | 10/16/2024 |
| Sara Castro[23] | 2011 | Peru | Cajamarca | Positive (PCR) or suspected cases | Viral Prevalence | 0 | 26 | 0.000 | RT-PCR | NR | Emilie Toews | 10/16/2024 |
| Belgath Fernandes Cardoso[24] | 2012 | Brazil | Mato Grosso | Febrile patients | Viral Prevalence | 5 | 524 | 0.010 | RT-PCR | S segment (OROV only) | Emilie Toews | 10/16/2024 |
| Michele S. Bastos[25] | 2012 | Brazil | Amazonas | Non-arboviral patients | Viral Prevalence | 6 | 330 | 0.018 | RT-PCR | S segment (OROV only) | Harriet Ware | 10/16/2024 |
| Vivaldo Gomes da Costa[26] | 2013 | Brazil | Goiás | Positive (PCR) or suspected cases | Seroprevalence | 10 | 1,294 | 0.008 | ELISA | IgM | Emilie Toews | 10/16/2024 |
| Maha Elbadry[27] | 2014 | Haiti |  | Febrile patients | Viral Prevalence | 1 | 1,250 | 0.001 | RT-PCR | NR | Emilie Toews | 10/16/2024 |
| Raquel Curtinhas de Lima[28] | 2015 | Brazil | Amapa | Febrile patients | Seroprevalence | 17 | 166 | 0.102 | PRNT | NAb | Harriet Ware | 10/16/2024 |
| Raquel Curtinhas de Lima[28] | 2015 | Brazil | Amapa | Febrile patients | Viral Prevalence | 0 | 166 | 0.000 | RT-PCR | S segment (OROV only) | Harriet Ware | 10/16/2024 |
| Felipe Naveca[29] | 2015 | Brazil | Amazonas | Febrile patients | Viral Prevalence | 9 | 30 | 0.300 | RT-PCR | S segment (OROV only) | Emilie Toews | 10/16/2024 |
| Barbara Batista Salgado[30] | 2015 | Brazil | Amazonas State | Essential non-healthcare workers | Seroprevalence | 4 | 595 | 0.007 | HAI | NAb | Sabah Shaikh | 10/10/2024 |
| Maria Garcia[31] | 2016 | Peru | Madre de Dios | Febrile patients | Seroprevalence | 122 | 508 | 0.240 | ELISA | IgM | Emilie Toews | 10/16/2024 |
| Maria Garcia[31] | 2016 | Peru | Madre de Dios | Febrile patients | Viral Prevalence | 51 | 1,016 | 0.050 | RT-PCR | NR | Emilie Toews | 10/16/2024 |
| Tung Gia Phan[32] | 2016 | Peru |  | Febrile patients | Viral Prevalence | 2 | 18 | 0.111 | RT-PCR | NR | Emilie Toews | 10/16/2024 |
| Carlos Alva-Urcia[33] | 2016 | Peru | Madre de Dios | Febrile patients | Viral Prevalence | 24 | 278 | 0.086 | RT-PCR | NR | Emilie Toews | 10/16/2024 |
| Helver Dias[34] | 2016 | Brazil | Mato Grosso do Sul | Febrile patients | Viral Prevalence | 0 | 106 | 0.000 | RT-PCR | S segment (OROV only) | Emilie Toews | 10/16/2024 |
| Valdinete Alves do Nascimento[35] | 2016 | Brazil | Amazonas | Positive (PCR) or suspected cases | Viral Prevalence | 5 | 352 | 0.014 | RT-PCR | NR | Emilie Toews | 10/16/2024 |
| Juana del Valle-Mendoza[36] | 2016 | Peru | Cajamarca | Febrile patients | Viral Prevalence | 0 | 248 | 0.000 | RT-PCR | NR | Emilie Toews | 10/16/2024 |
| Hilda Durango-Chavez[37] | 2016 | Peru |  | Febrile patients | Viral Prevalence | 151 | 741 | 0.204 | RT-PCR | NR | Emilie Toews | 10/16/2024 |
| M.C. de Souza Costa[38] | 2016 | Brazil | Mato Grosso | Febrile patients | Viral Prevalence | 40 | 897 | 0.045 | RT-PCR | S segment (OROV only) | Emilie Toews | 10/16/2024 |
| Wilmer Silva-Caso[39] | 2016 | Peru | Huanuco | Febrile patients | Viral Prevalence | 92 | 536 | 0.172 | RT-PCR | S segment (OROV only) | Emilie Toews | 10/16/2024 |
| Wilmer Silva-Caso[40] | 2016 | Peru | Huánuco | Febrile patients | Viral Prevalence | 46 | 268 | 0.172 | RT-PCR | NR | Caseng Zhang | 10/16/2024 |
| Johanna Martins-Luna[41] | 2016 | Peru | Piura | Febrile patients | Viral Prevalence | 131 | 496 | 0.264 | RT-PCR | NR | Emilie Toews | 10/16/2024 |
| Johanna Martins-Luna[42] | 2016 | Peru | NR | Febrile patients | Viral Prevalence | 393 | 1,488 | 0.264 | RT-PCR | NR | Sabah Shaikh | 10/16/2024 |
| Juana del Valle-Mendoza[43] | 2016 | Peru | Cajamarca | Febrile patients | Viral Prevalence | 0 | 95 | 0.000 | RT-PCR | NR | Emilie Toews | 10/16/2024 |
| Cassiano Junior Saatkamp[44] | 2016 | Brazil | Para | Febrile patients | Viral Prevalence | 0 | 49 | 0.000 | RT-PCR | S segment (OROV only) | Harriet Ware | 10/16/2024 |
| Emma L. Wise[45] | 2016 | Ecuador | Esmeraldas | Febrile patients | Viral Prevalence | 6 | 258 | 0.023 | RT-PCR | S segment (OROV only) | Emilie Toews | 10/16/2024 |
| Marco Coaguila[46] | 2017 | Peru |  | Febrile patients | Seroprevalence | 0 | 1,983 | 0.000 | Other | NR | Emilie Toews | 10/16/2024 |
| Juliana Gil-Mora[47] | 2017 | Colombia | Cauca | Community | Seroprevalence | 10 | 505 | 0.020 | PRNT | NAb | Caseng Zhang | 10/10/2024 |
| Larissa Moraes dos Santos Fonseca[48] | 2017 | Brazil | Bahia | Febrile patients | Viral Prevalence | 5 | 53 | 0.094 | RT-PCR | S segment (OROV only) | Sabah Shaikh | 10/16/2024 |
| Vanessa L. Carvalho[49] | 2018 | Brazil | Para State | Febrile patients | Seroprevalence | 37 | 90 | 0.411 | ELISA | IgM | Emilie Toews | 10/16/2024 |
| Janeth Aracely Ramirez Pavon[50] | 2019 | Brazil | Mato Grosso | Positive (PCR) or suspected cases | Viral Prevalence | 1 | 6 | 0.167 | RT-PCR | S segment (OROV only) | Emilie Toews | 10/16/2024 |
| Luiz Henrique Gonçalves Maciel[51] | 2019 | Brazil | Amazonas | Non-arboviral patients | Viral Prevalence | 0 | 340 | 0.000 | RT-PCR | S segment (OROV only) | Harriet Ware | 10/16/2024 |
| Jackson Alves da Silva Queiroz[52] | 2019 | Brazil | Rondonia | Febrile patients | Viral Prevalence | 0 | 308 | 0.000 | RT-PCR | NR | Emilie Toews | 10/16/2024 |
| Diego Michel Fernandes da Silva[53] | 2020 | Brazil | Goias | Positive (PCR) or suspected cases | Viral Prevalence | 0 | 79 | 0.000 | RT-PCR | NR | Emilie Toews | 10/16/2024 |
| Mélanie Gaillet[54] | 2020 | French Guiana |  | Febrile patients | Viral Prevalence | 23 | 28 | 0.821 | RT-PCR | NR | Sabah Shaikh | 10/16/2024 |
| Carlos Silva-Ramos[55] | 2021 | Colombia | Antioquia | Febrile patients | Viral Prevalence | 0 | 116 | 0.000 | RT-PCR | NR | Emilie Toews | 10/10/2024 |
| Karl Ciuoderis[56] | 2022 | Colombia |  | Febrile patients | Seroprevalence | 234 | 1,652 | 0.142 | MIA | IgG | Caseng Zhang | 10/10/2024 |
| Karl Ciuoderis[56] | 2022 | Colombia |  | Febrile patients | Viral Prevalence | 174 | 1,582 | 0.110 | RT-PCR | M segment (OROV only) | Caseng Zhang | 10/10/2024 |
| Hillquias Monteiro Moreira[57] | 2023 | Brazil | Rondonia and Amazonas | Febrile patients | Viral Prevalence | 27 | 351 | 0.077 | RT-PCR | S segment (OROV only),M segment (OROV only) | Caseng Zhang | 10/16/2024 |
| Liliana Sanchez-Lerma[58] | 2023 | Colombia | Meta | Febrile patients | Viral Prevalence | 0 | 100 | 0.000 | RT-PCR | N Segment | Emilie Toews | 10/16/2024 |
| Gabriel Scachetti[59] | 2024 | Brazil | Amazonas State | Febrile patients | Viral Prevalence | 10 | 93 | 0.108 | RT-PCR | NR | Emilie Toews | 10/16/2024 |

## Table B. Bibliographic summary of vector studies.

| Author | Sampling year | Country | State or province | Population | Species | Estimate type | Positive cases | Number tested | Prevalence (%) | Assay type | Assay target | Data extractor | Date extracted |
| --- | --- | --- | --- | --- | --- | --- | --- | --- | --- | --- | --- | --- | --- |
| F. P. Pinheiro[1] | 1975 | Brazil | Para | Non-human animal | Multiple | Seroprevalence | 47 | 1,494 | 0.031 | HAI | NAb | Emilie Toews | 10/16/2024 |
| F. P. Pinheiro[1] | 1975 | Brazil | Para | Insect | Culicoides paraensis | Viral Prevalence | 2 | 15,000 | 0.000 | Viral isolation | NAb | Emilie Toews | 10/16/2024 |
| Carlos Borborema[4] | 1980 | Brazil | Amazonas | Insect | Culex quinquefasciatus | Seroprevalence | 1 | 42 | 0.024 | HAI | NAb | Emilie Toews | 10/16/2024 |
| Carlos Borborema[4] | 1980 | Brazil | Amazonas | Insect | Culicoides paraensis | Seroprevalence | 0 | 147 | 0.000 | HAI | NAb | Emilie Toews | 10/16/2024 |
| Pedro Fernando da Costa Vasconcelos[5] | 1988 | Brazil | Maranhão | Insect | Aedes scapularis | Seroprevalence | 0 | 1 | 0.000 | CF | IgM,NAb | Shaila Akter | 10/16/2024 |
| Pedro Fernando da Costa Vasconcelos[5] | 1988 | Brazil | Maranhão | Insect | Anopheles nuneztovari | Seroprevalence | 0 | 1 | 0.000 | CF | IgM,NAb | Shaila Akter | 10/16/2024 |
| Pedro Fernando da Costa Vasconcelos[5] | 1988 | Brazil | Maranhão | Insect | Anopheles triannulatus | Seroprevalence | 0 | 1 | 0.000 | CF | IgM,NAb | Shaila Akter | 10/16/2024 |
| Pedro Fernando da Costa Vasconcelos[5] | 1988 | Brazil | Maranhão | Insect | Culex Carrolia Sp | Seroprevalence | 0 | 1 | 0.000 | CF | IgM,NAb | Shaila Akter | 10/16/2024 |
| Pedro Fernando da Costa Vasconcelos[5] | 1988 | Brazil | Maranhão | Insect | Culex Spp. | Seroprevalence | 0 | 1 | 0.000 | CF | IgM,NAb | Shaila Akter | 10/16/2024 |
| Pedro Fernando da Costa Vasconcelos[5] | 1988 | Brazil | Maranhão | Insect | Culex corniger | Seroprevalence | 0 | 1 | 0.000 | CF | IgM,NAb | Shaila Akter | 10/16/2024 |
| Pedro Fernando da Costa Vasconcelos[5] | 1988 | Brazil | Maranhão | Insect | Culex coronator | Seroprevalence | 0 | 1 | 0.000 | CF | IgM,NAb | Shaila Akter | 10/16/2024 |
| Pedro Fernando da Costa Vasconcelos[5] | 1988 | Brazil | Maranhão | Insect | Culex declarator | Seroprevalence | 0 | 3 | 0.000 | CF | IgM,NAb | Shaila Akter | 10/16/2024 |
| Pedro Fernando da Costa Vasconcelos[5] | 1988 | Brazil | Maranhão | Insect | Culex quinquefasciatus | Seroprevalence | 0 | 79 | 0.000 | CF | IgM,NAb | Shaila Akter | 10/16/2024 |
| Pedro Fernando da Costa Vasconcelos[5] | 1988 | Brazil | Maranhão | Insect | Culicoides paraensis | Seroprevalence | 1 | 39 | 0.026 | CF | IgM,NAb | Shaila Akter | 10/16/2024 |
| Pedro Fernando da Costa Vasconcelos[5] | 1988 | Brazil | Maranhão | Insect | Limatus Sp | Seroprevalence | 0 | 1 | 0.000 | CF | IgM,NAb | Shaila Akter | 10/16/2024 |
| Pedro Fernando da Costa Vasconcelos[5] | 1988 | Brazil | Maranhão | Insect | Mansonia Sp. | Seroprevalence | 0 | 1 | 0.000 | CF | IgM,NAb | Shaila Akter | 10/16/2024 |
| Pedro Fernando da Costa Vasconcelos[5] | 1988 | Brazil | Maranhão | Insect | Psorophora cingulata | Seroprevalence | 0 | 1 | 0.000 | CF | IgM,NAb | Shaila Akter | 10/16/2024 |
| Pedro Fernando da Costa Vasconcelos[5] | 1988 | Brazil | Maranhão | Insect | Psorophora ferox | Seroprevalence | 0 | 1 | 0.000 | CF | IgM,NAb | Shaila Akter | 10/16/2024 |
| Pedro Fernando da Costa Vasconcelos[5] | 1988 | Brazil | Maranhão | Insect | Sabethes glaucodaemon | Seroprevalence | 0 | 1 | 0.000 | CF | IgM,NAb | Shaila Akter | 10/16/2024 |
| Pedro Fernando da Costa Vasconcelos[5] | 1988 | Brazil | Maranhão | Insect | Uranotaenia Sp | Seroprevalence | 0 | 1 | 0.000 | CF | IgM,NAb | Shaila Akter | 10/16/2024 |
| Pedro Fernando da Costa Vasconcelos[5] | 1988 | Brazil | Maranhão | Insect | Wyeomiya Sp. | Seroprevalence | 0 | 1 | 0.000 | CF | IgM,NAb | Shaila Akter | 10/16/2024 |
| Scott Medlin[60] | 2007 | Costa Rica |  | Non-human animal | Bradypus variegatus | Seroprevalence | 0 | 94 | 0.000 | HAI | NAb | Emilie Toews | 10/16/2024 |
| Scott Medlin[60] | 2007 | Costa Rica |  | Non-human animal | Choloepus hoffmanni | Seroprevalence | 0 | 94 | 0.000 | HAI | NAb | Emilie Toews | 10/16/2024 |
| Ana Cecilia Ribeiro Cruz[19] | 2007 | Brazil | Pará | Non-human animal | Conopophage roberti | Seroprevalence | 0 | 1 | 0.000 | HAI | NAb | Emilie Toews | 10/16/2024 |
| Ana Cecilia Ribeiro Cruz[19] | 2007 | Brazil | Pará | Non-human animal | Didelphis marsupialis | Seroprevalence | 0 | 5 | 0.000 | HAI | NAb | Emilie Toews | 10/16/2024 |
| Ana Cecilia Ribeiro Cruz[19] | 2007 | Brazil | Pará | Non-human animal | Geotrygon montana | Seroprevalence | 0 | 1 | 0.000 | HAI | NAb | Emilie Toews | 10/16/2024 |
| Ana Cecilia Ribeiro Cruz[19] | 2007 | Brazil | Pará | Non-human animal | Myrmothera companisona | Seroprevalence | 0 | 1 | 0.000 | HAI | NAb | Emilie Toews | 10/16/2024 |
| Ana Cecilia Ribeiro Cruz[19] | 2007 | Brazil | Pará | Non-human animal | Phlegopsis n. pareensis | Seroprevalence | 0 | 72 | 0.000 | HAI | NAb | Emilie Toews | 10/16/2024 |
| Ana Cecilia Ribeiro Cruz[19] | 2007 | Brazil | Pará | Non-human animal | Proechimis guianensis | Seroprevalence | 0 | 2 | 0.000 | HAI | NAb | Emilie Toews | 10/16/2024 |
| Ana Cecilia Ribeiro Cruz[19] | 2007 | Brazil | Pará | Non-human animal | Schiffornis tudinus | Seroprevalence | 0 | 1 | 0.000 | HAI | NAb | Emilie Toews | 10/16/2024 |
| Ana Cecilia Ribeiro Cruz[19] | 2007 | Brazil | Pará | Non-human animal | Thamnophilus aethiops | Seroprevalence | 0 | 1 | 0.000 | HAI | NAb | Emilie Toews | 10/16/2024 |
| Ana Cecilia Ribeiro Cruz[19] | 2007 | Brazil | Pará | Non-human animal | Xiphorhynchus ocellatus | Seroprevalence | 0 | 1 | 0.000 | HAI | NAb | Emilie Toews | 10/16/2024 |
| Plautino O. Laroque[61] | 2010 | Brazil | Paraiba | Non-human animal | Cebus flavius | Seroprevalence | 0 | 31 | 0.000 | HAI | NAb | Emilie Toews | 10/16/2024 |
| Plautino O. Laroque[61] | 2010 | Brazil |  | Non-human animal | Cebus libidinosus | Seroprevalence | 28 | 100 | 0.280 | HAI | NAb | Emilie Toews | 10/16/2024 |
| Paulo Mira Batista[62] | 2010 | Brazil | Mato Grosso do Sul | Non-human animal | Multiple | Seroprevalence | 5 | 65 | 0.077 | HAI | NR | Shaila Akter | 10/16/2024 |
| Alex Pauvolid-Correa[63] | 2011 | Brazil | Mato Grosso do Sul | Non-human animal | Caiman species | Seroprevalence | 0 | 66 | 0.000 | PRNT | NAb | Emilie Toews | 10/16/2024 |
| Alex Pauvolid-Correa[63] | 2011 | Brazil | Mato Grosso do Sul | Non-human animal | Equine species | Seroprevalence | 0 | 375 | 0.000 | PRNT | NAb | Emilie Toews | 10/16/2024 |
| Alex Pauvolid-Correa[63] | 2011 | Brazil | Mato Grosso do Sul | Non-human animal | Sheep species | Seroprevalence | 1 | 232 | 0.004 | PRNT | NAb | Emilie Toews | 10/16/2024 |
| Michael J. Turell[64] | 2011 | Peru | Maynas | Non-human animal | Aotus nancymaae | Seroprevalence | 0 | 20 | 0.000 | ELISA | IgG,IgM | Emilie Toews | 10/16/2024 |
| Belgath Fernandes Cardoso[24] | 2012 | Brazil | Mato Grosso | Insect | Culex quinquefasciatus | Viral Prevalence | 8 | 387 | 0.021 | RT-PCR | S segment (OROV only) | Emilie Toews | 10/16/2024 |
| Paulo Mira Batista[65] | 2013 | Brazil | Mato Grosso do Sul | Non-human animal | Non-human primates | Seroprevalence | 3 | 48 | 0.062 | HAI | NAb | Emilie Toews | 10/16/2024 |
| Jordam Pereira-Silva[66] | 2016 | Brazil | Amazonas | Insect | Multiple | Viral Prevalence | 3 | 671 | 0.004 | RT-PCR | S segment (OROV only) | Emilie Toews | 10/16/2024 |
| Laura Tauro[67] | 2017 | Brazil | Bahia | Insect | Aedes aegypti | Viral Prevalence | 0 | 26 | 0.000 | RT-PCR | NR | Emilie Toews | 10/16/2024 |
| Laura Tauro[67] | 2017 | Brazil | Bahia | Insect | Culex quinquefasciatus | Viral Prevalence | 0 | 99 | 0.000 | RT-PCR | NR | Emilie Toews | 10/16/2024 |
| Raquel da Silva Ferreira[68] | 2018 | Brazil | Mato Grosso | Insect | Aedes aegypti | Viral Prevalence | 1 | 84 | 0.012 | RT-PCR | S segment (OROV only) | Emilie Toews | 10/16/2024 |
| Raquel da Silva Ferreira[68] | 2018 | Brazil | Mato Grosso | Insect | Culex Spp. | Viral Prevalence | 0 | 3 | 0.000 | RT-PCR | S segment (OROV only) | Emilie Toews | 10/16/2024 |
| Raquel da Silva Ferreira[68] | 2018 | Brazil | Mato Grosso | Insect | Culex quinquefasciatus | Viral Prevalence | 1 | 179 | 0.006 | RT-PCR | S segment (OROV only) | Emilie Toews | 10/16/2024 |
| Raquel da Silva Ferreira[68] | 2018 | Brazil | Mato Grosso | Insect | Psorophora albigenu | Viral Prevalence | 0 | 1 | 0.000 | RT-PCR | S segment (OROV only) | Emilie Toews | 10/16/2024 |
| Helver Gonçalves Dias[69] | 2018 | Brazil | Mato Grosso do Sul, Mato Grosso | Non-human animal | Bos indicus/taurus | Seroprevalence | 3 | 40 | 0.075 | PRNT | NAb | Harriet Ware | 10/16/2024 |
| Helver Gonçalves Dias[69] | 2018 | Brazil | Mato Grosso do Sul, Mato Grosso | Non-human animal | Canis lupus familiaris | Seroprevalence | 3 | 30 | 0.100 | PRNT | NAb | Harriet Ware | 10/16/2024 |
| Helver Gonçalves Dias[69] | 2018 | Brazil | Mato Grosso do Sul, Mato Grosso | Non-human animal | Equus ferus caballus | Seroprevalence | 0 | 35 | 0.000 | PRNT | NAb | Harriet Ware | 10/16/2024 |
| Helver Dias[34] | 2018 | Brazil | Mato Grosso | Insect | Aedes Spp. | Viral Prevalence | 0 | 62 | 0.000 | RT-PCR | S segment (OROV only) | Emilie Toews | 10/16/2024 |
| Helver Dias[34] | 2018 | Brazil | Mato Grosso | Insect | Aedes aegypti | Viral Prevalence | 0 | 280 | 0.000 | RT-PCR | S segment (OROV only) | Emilie Toews | 10/16/2024 |
| Helver Dias[34] | 2018 | Brazil | Mato Grosso | Insect | Aedes albopictus | Viral Prevalence | 0 | 94 | 0.000 | RT-PCR | S segment (OROV only) | Emilie Toews | 10/16/2024 |
| Helver Dias[34] | 2018 | Brazil | Mato Grosso | Insect | Aedes scapularis | Viral Prevalence | 0 | 101 | 0.000 | RT-PCR | S segment (OROV only) | Emilie Toews | 10/16/2024 |
| Helver Dias[34] | 2018 | Brazil | Mato Grosso do Sul | Insect | Culex Spp. | Viral Prevalence | 0 | 21,168 | 0.000 | RT-PCR | S segment (OROV only) | Emilie Toews | 10/16/2024 |
| Helver Dias[34] | 2018 | Brazil | Mato Grosso | Insect | Culex nigripalpus | Viral Prevalence | 0 | 175 | 0.000 | RT-PCR | S segment (OROV only) | Emilie Toews | 10/16/2024 |
| Helver Dias[34] | 2018 | Brazil | Mato Grosso | Insect | Culex quinquefasciatus | Viral Prevalence | 0 | 75 | 0.000 | RT-PCR | S segment (OROV only) | Emilie Toews | 10/16/2024 |
| Helver Dias[34] | 2018 | Brazil | Mato Grosso | Insect | Haemagogus Sp. | Viral Prevalence | 0 | 11 | 0.000 | RT-PCR | S segment (OROV only) | Emilie Toews | 10/16/2024 |
| Helver Dias[34] | 2018 | Brazil | Mato Grosso | Insect | Haemagogus janthinomys | Viral Prevalence | 0 | 52 | 0.000 | RT-PCR | S segment (OROV only) | Emilie Toews | 10/16/2024 |
| Helver Dias[34] | 2018 | Brazil | Mato Grosso | Insect | Haemagogus leucocelaenus | Viral Prevalence | 0 | 22 | 0.000 | RT-PCR | S segment (OROV only) | Emilie Toews | 10/16/2024 |
| Helver Dias[34] | 2018 | Brazil | Mato Grosso | Insect | Mansonia Sp. | Viral Prevalence | 0 | 34 | 0.000 | RT-PCR | S segment (OROV only) | Emilie Toews | 10/16/2024 |
| Helver Dias[34] | 2018 | Brazil | Mato Grosso; Mato Grosso do Sul | Insect | Multiple | Viral Prevalence | 0 | 22,931 | 0.000 | RT-PCR | S segment (OROV only) | Emilie Toews | 10/16/2024 |
| Helver Dias[34] | 2018 | Brazil | Mato Grosso | Insect | Psorophora Spp. | Viral Prevalence | 0 | 94 | 0.000 | RT-PCR | S segment (OROV only) | Emilie Toews | 10/16/2024 |
| Helver Dias[34] | 2018 | Brazil | Mato Grosso | Insect | Psorophora albigenu | Viral Prevalence | 0 | 134 | 0.000 | RT-PCR | S segment (OROV only) | Emilie Toews | 10/16/2024 |
| Helver Dias[34] | 2018 | Brazil | Mato Grosso | Insect | Psorophora cilipes | Viral Prevalence | 0 | 38 | 0.000 | RT-PCR | S segment (OROV only) | Emilie Toews | 10/16/2024 |
| Helver Dias[34] | 2018 | Brazil | Mato Grosso | Insect | Psorophora cingulata | Viral Prevalence | 0 | 39 | 0.000 | RT-PCR | S segment (OROV only) | Emilie Toews | 10/16/2024 |
| Helver Dias[34] | 2018 | Brazil | Mato Grosso | Insect | Psorophora dimidiata | Viral Prevalence | 0 | 191 | 0.000 | RT-PCR | S segment (OROV only) | Emilie Toews | 10/16/2024 |
| Helver Dias[34] | 2018 | Brazil | Mato Grosso | Insect | Psorophora lanei | Viral Prevalence | 0 | 32 | 0.000 | RT-PCR | S segment (OROV only) | Emilie Toews | 10/16/2024 |
| Helver Dias[34] | 2018 | Brazil | Mato Grosso | Insect | Sabethes Spp. | Viral Prevalence | 0 | 16 | 0.000 | RT-PCR | S segment (OROV only) | Emilie Toews | 10/16/2024 |
| Helver Dias[34] | 2018 | Brazil | Mato Grosso | Insect | Wyeomiya Sp. | Viral Prevalence | 0 | 313 | 0.000 | RT-PCR | S segment (OROV only) | Emilie Toews | 10/16/2024 |
| Helver Dias[34] | 2018 | Brazil | Mato Grosso do Sul | Non-human animal | Alouatta caraya | Viral Prevalence | 0 | 3 | 0.000 | RT-PCR | S segment (OROV only) | Emilie Toews | 10/16/2024 |
| Helver Dias[34] | 2018 | Brazil | Mato Grosso | Non-human animal | Aotus lemurinus | Viral Prevalence | 0 | 2 | 0.000 | RT-PCR | S segment (OROV only) | Emilie Toews | 10/16/2024 |
| Helver Dias[34] | 2018 | Brazil | Mato Grosso | Non-human animal | Ateles marginatus | Viral Prevalence | 0 | 2 | 0.000 | RT-PCR | S segment (OROV only) | Emilie Toews | 10/16/2024 |
| Helver Dias[34] | 2018 | Brazil | Mato Grosso | Non-human animal | Bos indicus/taurus | Viral Prevalence | 0 | 176 | 0.000 | RT-PCR | S segment (OROV only) | Emilie Toews | 10/16/2024 |
| Helver Dias[34] | 2018 | Brazil | Mato Grosso do Sul | Non-human animal | Callithrix jacchus | Viral Prevalence | 0 | 4 | 0.000 | RT-PCR | S segment (OROV only) | Emilie Toews | 10/16/2024 |
| Helver Dias[34] | 2018 | Brazil | Mato Grosso | Non-human animal | Canis lupus familiaris | Viral Prevalence | 0 | 174 | 0.000 | RT-PCR | S segment (OROV only) | Emilie Toews | 10/16/2024 |
| Helver Dias[34] | 2018 | Brazil | Mato Grosso | Non-human animal | Didelphis albiventris | Viral Prevalence | 0 | 73 | 0.000 | RT-PCR | S segment (OROV only) | Emilie Toews | 10/16/2024 |
| Helver Dias[34] | 2018 | Brazil | Mato Grosso | Non-human animal | Didelphis aurita | Viral Prevalence | 0 | 3 | 0.000 | RT-PCR | S segment (OROV only) | Emilie Toews | 10/16/2024 |
| Helver Dias[34] | 2018 | Brazil | Mato Grosso | Non-human animal | Equus ferus caballus | Viral Prevalence | 0 | 160 | 0.000 | RT-PCR | S segment (OROV only) | Emilie Toews | 10/16/2024 |
| Helver Dias[34] | 2018 | Brazil | Mato Grosso | Non-human animal | Felis silvestris catus | Viral Prevalence | 0 | 85 | 0.000 | RT-PCR | S segment (OROV only) | Emilie Toews | 10/16/2024 |
| Helver Dias[34] | 2018 | Brazil | Mato Grosso | Non-human animal | Mico melanurus | Viral Prevalence | 0 | 29 | 0.000 | RT-PCR | S segment (OROV only) | Emilie Toews | 10/16/2024 |
| Helver Dias[34] | 2018 | Brazil | Mato Grosso | Non-human animal | Nasua nasua | Viral Prevalence | 0 | 83 | 0.000 | RT-PCR | S segment (OROV only) | Emilie Toews | 10/16/2024 |
| Helver Dias[34] | 2018 | Brazil | Mato Grosso | Non-human animal | Sapajus apella | Viral Prevalence | 0 | 5 | 0.000 | RT-PCR | S segment (OROV only) | Emilie Toews | 10/16/2024 |
| Helver Dias[34] | 2018 | Brazil | Mato Grosso do Sul | Non-human animal | Sapajus cay | Viral Prevalence | 0 | 11 | 0.000 | RT-PCR | S segment (OROV only) | Emilie Toews | 10/16/2024 |
| Luiz Henrique Maciel Feitoza[70] | 2020 | Brazil | Rondônia | Insect | Culicoides paraensis | Viral Prevalence | 0 | 271 | 0.000 | RT-PCR | S segment (OROV only) | Sabah Shaikh | 10/16/2024 |
| Diego Michel Fernandes da Silva[71] | 2022 | Brazil | Goias | Insect | Aedes aegypti | Viral Prevalence | 0 | 1,570 | 0.000 | RT-PCR | NR | Emilie Toews | 10/16/2024 |

## Table C. Risk of bias breakdown for all studies.

| **Author** | **Grouping Variable** | **Study Population (OROV only)** | **Study Species (OROV only)** | **Estimate Type** | **Item 1 (JBI-M)** | **Item 5 (JBI-M)** | **Item 2 (JBI-A)** | **Item 3 (JBI-A)** | **Item 4 (JBI-A)** | **Item 6 (JBI-A)** | **Item 7 (JBI-A)** | **Item 8B (JBI-A)** | **Item 8A (JBI-A)** | **JBI-A Outputs** |
| --- | --- | --- | --- | --- | --- | --- | --- | --- | --- | --- | --- | --- | --- | --- |
| Pedro P. Alvarez | Overall | Human | Homo sapiens | Seroprevalence | Yes | Unclear | No | Yes | Yes | Yes | Yes | No | Yes | Moderate |
| Juana del Valle-Mendoza | Overall | Human | Homo sapiens | Viral Prevalence | No | Unclear | No | No | No | Yes | Yes | No | Yes | High |
| F. P. Pinheiro | Species | Human | Homo sapiens | Seroprevalence | Yes | Unclear | No | Yes | Yes | Yes | Yes | No | Yes | Moderate |
| Brett Forshey | Geography | Human | Homo sapiens | Viral Prevalence | Yes | Yes | Yes | Yes | Yes | Yes | Yes | Yes | Yes | Low |
| Brett Forshey | Geography | Human | Homo sapiens | Viral Prevalence | Yes | Yes | Yes | Yes | Yes | Yes | Yes | Yes | Yes | Low |
| Brett Forshey | Geography | Human | Homo sapiens | Viral Prevalence | Yes | Yes | Yes | Yes | Yes | Yes | Yes | Yes | Yes | Low |
| Brett Forshey | Geography | Human | Homo sapiens | Viral Prevalence | Yes | Yes | Yes | Yes | Yes | Yes | Yes | Yes | Yes | Low |
| Brett Forshey | Geography | Human | Homo sapiens | Viral Prevalence | Yes | Yes | Yes | Yes | Yes | Yes | Yes | Yes | Yes | Low |
| Brett Forshey | Geography | Human | Homo sapiens | Viral Prevalence | Yes | Yes | Yes | Yes | Yes | Yes | Yes | Yes | Yes | Low |
| Brett Forshey | Geography | Human | Homo sapiens | Viral Prevalence | Yes | Yes | Yes | Yes | Yes | Yes | Yes | Yes | Yes | Low |
| Brett Forshey | Geography | Human | Homo sapiens | Viral Prevalence | Yes | Yes | Yes | Yes | Yes | Yes | Yes | Yes | Yes | Low |
| Brett Forshey | Geography | Human | Homo sapiens | Viral Prevalence | Yes | Yes | Yes | Yes | Yes | Yes | Yes | Yes | Yes | Low |
| Brett Forshey | Geography | Human | Homo sapiens | Viral Prevalence | Yes | Yes | Yes | Yes | Yes | Yes | Yes | Yes | Yes | Low |
| Brett Forshey | Geography | Human | Homo sapiens | Viral Prevalence | Yes | Yes | Yes | Yes | Yes | Yes | Yes | Yes | Yes | Low |
| Brett Forshey | Geography | Human | Homo sapiens | Viral Prevalence | Yes | Yes | Yes | Yes | Yes | Yes | Yes | Yes | Yes | Low |
| Brett Forshey | Geography | Human | Homo sapiens | Viral Prevalence | Yes | Yes | Yes | Yes | Yes | Yes | Yes | Yes | Yes | Low |
| Brett Forshey | Gender | Human | Homo sapiens | Viral Prevalence | Yes | Yes | Yes | Yes | Yes | Yes | Yes | Yes | Yes | Low |
| Brett Forshey | Gender | Human | Homo sapiens | Viral Prevalence | Yes | Yes | Yes | Yes | Yes | Yes | Yes | Yes | Yes | Low |
| Juliana Gil-Mora | Geography | Human | Homo sapiens | Seroprevalence | Yes | No | Yes | Yes | Yes | Yes | Yes | Yes | Yes | Low |
| Juliana Gil-Mora | Geography | Human | Homo sapiens | Seroprevalence | Yes | No | Yes | Yes | Yes | Yes | Yes | Yes | Yes | Low |
| Juliana Gil-Mora | Geography | Human | Homo sapiens | Seroprevalence | Yes | No | Yes | No | Yes | Yes | Yes | Yes | Yes | Moderate |
| Juliana Gil-Mora | Geography | Human | Homo sapiens | Seroprevalence | Yes | No | Yes | No | Yes | Yes | Yes | Yes | Yes | Moderate |
| M.C. de Souza Costa | Gender | Human | Homo sapiens | Viral Prevalence | Yes | No | Yes | Yes | Yes | Yes | Yes | Yes | Yes | Low |
| M.C. de Souza Costa | Gender | Human | Homo sapiens | Viral Prevalence | Yes | No | Yes | Yes | Yes | Yes | Yes | Yes | Yes | Low |
| Vanessa L. Carvalho | Overall | Human | Homo sapiens | Seroprevalence | No | Unclear | Yes | No | No | Yes | Yes | No | Yes | High |
| Sara Castro | Test type | Human | Homo sapiens | Viral Prevalence | Yes | No | Yes | No | Yes | Yes | Yes | Yes | Yes | Moderate |
| Sara Castro | Test type | Human | Homo sapiens | Seroprevalence | Yes | No | Yes | No | Yes | Yes | Yes | Yes | Yes | Moderate |
| Valquiria do Carmo Alves Martins | Overall | Human | Homo sapiens | Viral Prevalence | No | Yes | Yes | Yes | Yes | Yes | Yes | No | Yes | Moderate |
| Maria Garcia | Overall | Human | Homo sapiens | Seroprevalence | Yes | Yes | Yes | Yes | No | Yes | Yes | Yes | Yes | Low |
| Maria Garcia | Overall | Human | Homo sapiens | Viral Prevalence | Yes | Yes | Yes | Yes | No | Yes | Yes | Yes | Yes | Low |
| Maria Garcia | Overall | Human | Homo sapiens | Viral Prevalence | Yes | Yes | Yes | Yes | No | Yes | Yes | Yes | Yes | Low |
| Michele S. Bastos | Overall | Human | Homo sapiens | Viral Prevalence | Yes | Yes | Yes | Yes | Yes | Yes | Yes | Yes | Yes | Low |
| Belgath Fernandes Cardoso | Gender | Human | Homo sapiens | Viral Prevalence | No | Yes | No | Yes | Yes | Yes | Yes | No | Yes | High |
| Belgath Fernandes Cardoso | Gender | Human | Homo sapiens | Viral Prevalence | No | Yes | No | Yes | Yes | Yes | Yes | No | Yes | High |
| Regina Maria Pinto De Figueiredo | Overall | Human | Homo sapiens | Seroprevalence | No | Yes | Yes | No | Yes | Yes | Yes | No | Yes | High |
| Marcio Nunes | Timeframe | Human | Homo sapiens | Seroprevalence | No | Unclear | No | Yes | No | Yes | Yes | No | Yes | High |
| Marcio Nunes | Timeframe | Human | Homo sapiens | Seroprevalence | No | Unclear | No | Yes | No | Yes | Yes | No | Yes | High |
| Marcio Nunes | Timeframe | Human | Homo sapiens | Seroprevalence | No | Unclear | No | Yes | No | Yes | Yes | No | Yes | High |
| Marcio Nunes | Timeframe | Human | Homo sapiens | Seroprevalence | No | Unclear | No | Yes | No | Yes | Yes | No | Yes | High |
| Marcio Nunes | Timeframe | Human | Homo sapiens | Seroprevalence | No | Unclear | No | Yes | No | Yes | Yes | No | Yes | High |
| Carlos Alva-Urcia | Gender | Human | Homo sapiens | Viral Prevalence | Yes | No | Yes | No | Yes | Yes | Yes | Yes | Yes | Moderate |
| Carlos Alva-Urcia | Gender | Human | Homo sapiens | Viral Prevalence | Yes | No | Yes | No | Yes | Yes | Yes | Yes | Yes | Moderate |
| Amélia Rosa | Gender | Human | Homo sapiens | Seroprevalence | Yes | Yes | Yes | Yes | Yes | Yes | Yes | Yes | Yes | Low |
| Amélia Rosa | Gender | Human | Homo sapiens | Seroprevalence | Yes | Yes | Yes | Yes | Yes | Yes | Yes | Yes | Yes | Low |
| Ronaldo B. Freitas | Gender | Human | Homo sapiens | Seroprevalence | Yes | Unclear | Yes | Yes | Yes | Yes | Yes | Yes | Yes | Low |
| Ronaldo B. Freitas | Gender | Human | Homo sapiens | Seroprevalence | Yes | Unclear | Yes | Yes | Yes | Yes | Yes | Yes | Yes | Low |
| Ronaldo B. Freitas | Gender | Human | Homo sapiens | Seroprevalence | Yes | Unclear | Yes | Yes | Yes | Yes | Yes | Yes | Yes | Low |
| Ronaldo B. Freitas | Gender | Human | Homo sapiens | Seroprevalence | Yes | Unclear | Yes | Yes | Yes | Yes | Yes | Yes | Yes | Low |
| Ronaldo B. Freitas | Gender | Human | Homo sapiens | Seroprevalence | Yes | Unclear | Yes | Yes | Yes | Yes | Yes | Yes | Yes | Low |
| Ronaldo B. Freitas | Gender | Human | Homo sapiens | Seroprevalence | Yes | Unclear | Yes | Yes | Yes | Yes | Yes | Yes | Yes | Low |
| Ronaldo B. Freitas | Gender | Human | Homo sapiens | Seroprevalence | Yes | Unclear | Yes | No | Yes | Yes | Yes | Yes | Yes | Moderate |
| Ronaldo B. Freitas | Gender | Human | Homo sapiens | Seroprevalence | Yes | Unclear | Yes | No | Yes | Yes | Yes | Yes | Yes | Moderate |
| Ronaldo B. Freitas | Gender | Human | Homo sapiens | Seroprevalence | Yes | Unclear | Yes | No | Yes | Yes | Yes | Yes | Yes | Moderate |
| Ronaldo B. Freitas | Gender | Human | Homo sapiens | Seroprevalence | Yes | Unclear | Yes | No | Yes | Yes | Yes | Yes | Yes | Moderate |
| Ronaldo B. Freitas | Gender | Human | Homo sapiens | Seroprevalence | Yes | Unclear | Yes | Yes | Yes | Yes | Yes | Yes | Yes | Low |
| Ronaldo B. Freitas | Gender | Human | Homo sapiens | Seroprevalence | Yes | Unclear | Yes | Yes | Yes | Yes | Yes | Yes | Yes | Low |
| Ronaldo B. Freitas | Gender | Human | Homo sapiens | Seroprevalence | Yes | Unclear | Yes | No | Yes | Yes | Yes | Yes | Yes | Moderate |
| Ronaldo B. Freitas | Gender | Human | Homo sapiens | Seroprevalence | Yes | Unclear | Yes | No | Yes | Yes | Yes | Yes | Yes | Moderate |
| Ronaldo B. Freitas | Test type | Human | Homo sapiens | Seroprevalence | Yes | Unclear | Yes | Yes | Yes | Yes | Yes | Yes | Yes | Low |
| Ronaldo B. Freitas | Test type | Human | Homo sapiens | Viral Prevalence | Yes | Unclear | Yes | Yes | Yes | Yes | Yes | Yes | Yes | Low |
| Ronaldo B. Freitas | Test type | Human | Homo sapiens | Seroprevalence | Yes | Unclear | Yes | No | Yes | Yes | Yes | Yes | Yes | Moderate |
| Ronaldo B. Freitas | Test type | Human | Homo sapiens | Viral Prevalence | Yes | Unclear | Yes | No | Yes | Yes | Yes | Yes | Yes | Moderate |
| Ronaldo B. Freitas | Test type | Human | Homo sapiens | Seroprevalence | Yes | Unclear | Yes | No | Yes | Yes | Yes | Yes | Yes | Moderate |
| Ronaldo B. Freitas | Test type | Human | Homo sapiens | Viral Prevalence | Yes | Unclear | Yes | No | Yes | Yes | Yes | Yes | Yes | Moderate |
| Ronaldo B. Freitas | Test type | Human | Homo sapiens | Seroprevalence | Yes | Unclear | Yes | No | Yes | Yes | Yes | Yes | Yes | Moderate |
| Ronaldo B. Freitas | Test type | Human | Homo sapiens | Viral Prevalence | Yes | Unclear | Yes | No | Yes | Yes | Yes | Yes | Yes | Moderate |
| Ronaldo B. Freitas | Test type | Human | Homo sapiens | Seroprevalence | Yes | Unclear | Yes | No | Yes | Yes | Yes | Yes | Yes | Moderate |
| Ronaldo B. Freitas | Test type | Human | Homo sapiens | Viral Prevalence | Yes | Unclear | Yes | No | Yes | Yes | Yes | Yes | Yes | Moderate |
| Ronaldo B. Freitas | Test type | Human | Homo sapiens | Seroprevalence | Yes | Unclear | Yes | Yes | Yes | Yes | Yes | Yes | Yes | Low |
| Ronaldo B. Freitas | Test type | Human | Homo sapiens | Viral Prevalence | Yes | Unclear | Yes | Yes | Yes | Yes | Yes | Yes | Yes | Low |
| Ronaldo B. Freitas | Test type | Human | Homo sapiens | Seroprevalence | Yes | Unclear | Yes | No | Yes | Yes | Yes | Yes | Yes | Moderate |
| Ronaldo B. Freitas | Test type | Human | Homo sapiens | Viral Prevalence | Yes | Unclear | Yes | No | Yes | Yes | Yes | Yes | Yes | Moderate |
| Ronaldo B. Freitas | Test type | Human | Homo sapiens | Seroprevalence | Yes | Unclear | Yes | No | Yes | Yes | Yes | Yes | Yes | Moderate |
| Ronaldo B. Freitas | Test type | Human | Homo sapiens | Viral Prevalence | Yes | Unclear | Yes | No | Yes | Yes | Yes | Yes | Yes | Moderate |
| Ronaldo B. Freitas | Test type | Human | Homo sapiens | Seroprevalence | Yes | Unclear | Yes | No | Yes | Yes | Yes | Yes | Yes | Moderate |
| Ronaldo B. Freitas | Test type | Human | Homo sapiens | Viral Prevalence | Yes | Unclear | Yes | No | Yes | Yes | Yes | Yes | Yes | Moderate |
| James LeDuc | Timeframe | Human | Homo sapiens | Seroprevalence | No | Yes | No | No | Yes | Yes | Yes | No | Yes | High |
| James LeDuc | Timeframe | Human | Homo sapiens | Seroprevalence | No | Yes | No | Yes | Yes | Yes | Yes | No | Yes | High |
| James LeDuc | Timeframe | Human | Homo sapiens | Seroprevalence | No | Yes | No | Yes | Yes | Yes | Yes | No | Yes | High |
| Kathy Baisley | Gender | Human | Homo sapiens | Seroprevalence | Yes | Yes | Yes | Yes | Yes | Yes | Yes | Yes | Yes | Low |
| Kathy Baisley | Gender | Human | Homo sapiens | Seroprevalence | Yes | Yes | Yes | Yes | Yes | Yes | Yes | Yes | Yes | Low |
| Carlos Silva-Ramos | Overall | Human | Homo sapiens | Viral Prevalence | Yes | No | Yes | No | Yes | Yes | Yes | Yes | Yes | Moderate |
| Carlos Silva-Ramos | Overall | Human | Homo sapiens | Viral Prevalence | Yes | No | Yes | No | Yes | Yes | Yes | Yes | Yes | Moderate |
| Douglas M. Watts | Overall | Human | Homo sapiens | Seroprevalence | Yes | Yes | Yes | Yes | Yes | Yes | Yes | Yes | Yes | Low |
| Douglas M. Watts | Gender | Human | Homo sapiens | Seroprevalence | Yes | Yes | Yes | Yes | Yes | Yes | Yes | Yes | Yes | Low |
| Douglas M. Watts | Gender | Human | Homo sapiens | Seroprevalence | Yes | Yes | Yes | Yes | Yes | Yes | Yes | Yes | Yes | Low |
| Stephen Manock | Test type | Human | Homo sapiens | Seroprevalence | No | Yes | Yes | Yes | Yes | Yes | Yes | No | Yes | Moderate |
| Stephen Manock | Test type | Human | Homo sapiens | Seroprevalence | No | Yes | Yes | Yes | Yes | Yes | Yes | No | Yes | Moderate |
| Wilmer Silva-Caso | Gender | Human | Homo sapiens | Viral Prevalence | No | Yes | No | Yes | Yes | Yes | Yes | No | Yes | High |
| Wilmer Silva-Caso | Gender | Human | Homo sapiens | Viral Prevalence | No | Yes | No | Yes | Yes | Yes | Yes | No | Yes | High |
| Felipe Naveca | Overall | Human | Homo sapiens | Viral Prevalence | Yes | Unclear | Yes | No | No | Yes | Yes | Yes | Yes | Moderate |
| Cassiano Junior Saatkamp | Overall | Human | Homo sapiens | Viral Prevalence | Yes | Unclear | Yes | No | Yes | Yes | Yes | Yes | Yes | Moderate |
| Janeth Aracely Ramirez Pavon | Overall | Human | Homo sapiens | Viral Prevalence | Yes | No | Yes | No | Yes | Yes | Yes | Yes | Yes | Moderate |
| Helena Vasconcelos | Test type | Human | Homo sapiens | Seroprevalence | No | Unclear | Yes | Yes | Yes | Yes | Yes | No | Yes | Moderate |
| Helena Vasconcelos | Test type | Human | Homo sapiens | Seroprevalence | No | Unclear | Yes | Yes | Yes | Yes | Yes | No | Yes | Moderate |
| Helena Vasconcelos | Geography | Human | Homo sapiens | Seroprevalence | No | Unclear | Yes | Yes | Yes | Yes | Yes | No | Yes | Moderate |
| Helena Vasconcelos | Geography | Human | Homo sapiens | Seroprevalence | No | Unclear | Yes | No | Yes | Yes | Yes | No | Yes | High |
| Maria Paula Mourão | Overall | Human | Homo sapiens | Seroprevalence | Yes | No | Yes | Yes | Yes | Yes | Yes | Yes | Yes | Low |
| Johanna Martins-Luna | Overall | Human | Homo sapiens | Viral Prevalence | Yes | Yes | Yes | Yes | Yes | Yes | Yes | Yes | Yes | Low |
| Johanna Martins-Luna | Gender | Human | Homo sapiens | Viral Prevalence | Yes | Yes | Yes | Yes | Yes | Yes | Yes | Yes | Yes | Low |
| Johanna Martins-Luna | Gender | Human | Homo sapiens | Viral Prevalence | Yes | Yes | Yes | Yes | Yes | Yes | Yes | Yes | Yes | Low |
| Karl Ciuoderis | Test type | Human | Homo sapiens | Viral Prevalence | Yes | No | Yes | Yes | Yes | Yes | Yes | Yes | Yes | Low |
| Karl Ciuoderis | Test type | Human | Homo sapiens | Seroprevalence | Yes | No | Yes | Yes | Yes | Yes | Yes | Yes | Yes | Low |
| Karl Ciuoderis | Test type | Human | Homo sapiens | Seroprevalence | Yes | No | Yes | Yes | Yes | Yes | Yes | Yes | Yes | Low |
| Karl Ciuoderis | Test type | Human | Homo sapiens | Viral Prevalence | Yes | No | Yes | No | Yes | Yes | Yes | Yes | Yes | Moderate |
| Karl Ciuoderis | Test type | Human | Homo sapiens | Seroprevalence | Yes | No | Yes | No | Yes | Yes | Yes | Yes | Yes | Moderate |
| Karl Ciuoderis | Test type | Human | Homo sapiens | Viral Prevalence | Yes | No | Yes | No | Yes | Yes | Yes | Yes | Yes | Moderate |
| Karl Ciuoderis | Test type | Human | Homo sapiens | Seroprevalence | Yes | No | Yes | No | Yes | Yes | Yes | Yes | Yes | Moderate |
| Karl Ciuoderis | Test type | Human | Homo sapiens | Viral Prevalence | Yes | No | Yes | Yes | Yes | Yes | Yes | Yes | Yes | Low |
| Karl Ciuoderis | Test type | Human | Homo sapiens | Seroprevalence | Yes | No | Yes | Yes | Yes | Yes | Yes | Yes | Yes | Low |
| Karl Ciuoderis | Test type | Human | Homo sapiens | Viral Prevalence | Yes | No | Yes | Yes | Yes | Yes | Yes | Yes | Yes | Low |
| Karl Ciuoderis | Test type | Human | Homo sapiens | Seroprevalence | Yes | No | Yes | Yes | Yes | Yes | Yes | Yes | Yes | Low |
| Emma L. Wise | Overall | Human | Homo sapiens | Viral Prevalence | No | Unclear | Yes | Yes | Yes | Yes | Yes | No | Yes | Moderate |
| Emma L. Wise | Overall | Human | Homo sapiens | Viral Prevalence | No | Unclear | Yes | No | Yes | Yes | Yes | No | Yes | High |
| Larissa Moraes dos Santos Fonseca | Overall | Human | Homo sapiens | Viral Prevalence | Yes | Unclear | Yes | No | No | Yes | Yes | Yes | Yes | Moderate |
| Valdinete Alves do Nascimento | Overall | Human | Homo sapiens | Viral Prevalence | Yes | Unclear | Yes | Yes | No | Yes | Yes | Yes | Yes | Low |
| Raquel Curtinhas de Lima | Overall | Human | Homo sapiens | Seroprevalence | Yes | Yes | Yes | Yes | Yes | Yes | Yes | Yes | Yes | Low |
| Raquel Curtinhas de Lima | Overall | Human | Homo sapiens | Viral Prevalence | Yes | Yes | Yes | Yes | Yes | Yes | Yes | Yes | Yes | Low |
| Hilda Durango-Chavez | Overall | Human | Homo sapiens | Viral Prevalence | Yes | Yes | Yes | Yes | Yes | Yes | Yes | Yes | Yes | Low |
| Wilmer Silva-Caso | Overall | Human | Homo sapiens | Viral Prevalence | Yes | Unclear | Yes | Yes | No | Yes | Yes | Yes | Yes | Low |
| Johanna Martins-Luna | Overall | Human | Homo sapiens | Viral Prevalence | Yes | Unclear | Yes | Yes | No | Yes | Yes | Yes | Yes | Low |
| Maha Elbadry | Overall | Human | Homo sapiens | Viral Prevalence | Yes | Unclear | Yes | Yes | Yes | Yes | Yes | Yes | Yes | Low |
| Mélanie Gaillet | Overall | Human | Homo sapiens | Viral Prevalence | No | Yes | Yes | No | Yes | Yes | Yes | No | Yes | High |
| Hillquias Monteiro Moreira | Overall | Human | Homo sapiens | Viral Prevalence | Yes | Unclear | Yes | Yes | No | Yes | Yes | Yes | Yes | Low |
| José Tavares-Neto | Overall | Human | Homo sapiens | Seroprevalence | No | Unclear | No | Yes | Yes | Yes | Yes | No | Yes | High |
| José Tavares-Neto | Overall | Human | Homo sapiens | Seroprevalence | No | Unclear | Yes | Yes | Yes | Yes | Yes | No | Yes | Moderate |
| Jackson Alves da Silva Queiroz | Overall | Human | Homo sapiens | Viral Prevalence | Yes | No | Yes | Yes | No | Yes | Yes | Yes | Yes | Low |
| Barbara Batista Salgado | Overall | Human | Homo sapiens | Seroprevalence | No | No | Yes | Yes | Yes | Yes | Yes | No | Yes | Moderate |
| Luiz Henrique Gonçalves Maciel | Overall | Human | Homo sapiens | Viral Prevalence | Yes | Yes | Yes | Yes | Yes | Yes | Yes | Yes | Yes | Low |
| Carlos Borborema | Geography | Human | Homo sapiens | Seroprevalence | Yes | Unclear | Yes | No | No | Yes | Yes | Yes | Yes | Moderate |
| Carlos Borborema | Geography | Human | Homo sapiens | Seroprevalence | Yes | Unclear | Yes | No | No | Yes | Yes | Yes | Yes | Moderate |
| Carlos Borborema | Geography | Human | Homo sapiens | Seroprevalence | Yes | Unclear | Yes | No | No | Yes | Yes | Yes | Yes | Moderate |
| Carlos Borborema | Geography | Human | Homo sapiens | Seroprevalence | Yes | Unclear | Yes | Yes | No | Yes | Yes | Yes | Yes | Low |
| Carlos Borborema | Geography | Human | Homo sapiens | Seroprevalence | Yes | Unclear | Yes | No | No | Yes | Yes | Yes | Yes | Moderate |
| Carlos Borborema | Geography | Human | Homo sapiens | Seroprevalence | Yes | Unclear | Yes | No | No | Yes | Yes | Yes | Yes | Moderate |
| Carlos Borborema | Geography | Human | Homo sapiens | Seroprevalence | Yes | Unclear | Yes | No | No | Yes | Yes | Yes | Yes | Moderate |
| Carlos Borborema | Geography | Human | Homo sapiens | Seroprevalence | Yes | Unclear | Yes | No | No | Yes | Yes | Yes | Yes | Moderate |
| Carlos Borborema | Geography | Human | Homo sapiens | Seroprevalence | Yes | Unclear | Yes | No | No | Yes | Yes | Yes | Yes | Moderate |
| Carlos Borborema | Geography | Human | Homo sapiens | Seroprevalence | Yes | Unclear | Yes | Yes | No | Yes | Yes | Yes | Yes | Low |
| Carlos Borborema | Geography | Human | Homo sapiens | Seroprevalence | Yes | Unclear | Yes | No | No | Yes | Yes | Yes | Yes | Moderate |
| Carlos Borborema | Geography | Human | Homo sapiens | Seroprevalence | Yes | Unclear | Yes | No | No | Yes | Yes | Yes | Yes | Moderate |
| Carlos Borborema | Geography | Human | Homo sapiens | Seroprevalence | Yes | Unclear | No | No | No | Yes | Yes | No | Yes | High |
| Carlos Borborema | Geography | Human | Homo sapiens | Seroprevalence | Yes | Unclear | No | No | No | Yes | Yes | No | Yes | High |
| Pedro Fernando da Costa Vasconcelos | Gender | Human | Homo sapiens | Seroprevalence | Unclear | Yes | Yes | Yes | Yes | Yes | Yes | No | Yes | High |
| Pedro Fernando da Costa Vasconcelos | Gender | Human | Homo sapiens | Seroprevalence | Unclear | Yes | Yes | No | Yes | Yes | Yes | No | Yes | High |
| Diego Michel Fernandes da Silva | Overall | Human | Homo sapiens | Viral Prevalence | Yes | Unclear | Yes | No | Yes | Yes | Yes | Yes | Yes | Moderate |
| Marco Coaguila | Overall | Human | Homo sapiens | Seroprevalence | Yes | Unclear | Yes | Yes | No | Yes | Yes | Yes | Yes | Low |
| Raimunda do Socorro da Silva Azevedo | Gender | Human | Homo sapiens | Seroprevalence | Yes | Yes | No | No | Yes | Yes | Yes | No | Yes | High |
| Raimunda do Socorro da Silva Azevedo | Gender | Human | Homo sapiens | Seroprevalence | Yes | Yes | No | No | Yes | Yes | Yes | No | Yes | High |
| Raimunda do Socorro da Silva Azevedo | Overall | Human | Homo sapiens | Seroprevalence | Yes | Yes | No | Yes | Yes | Yes | Yes | No | Yes | Moderate |
| Gabriel Scachetti | Overall | Human | Homo sapiens | Viral Prevalence | No | Unclear | No | No | No | Yes | Yes | No | Yes | High |
| Helver Dias | Species | Human | Homo sapiens | Viral Prevalence | Yes | No | Yes | Yes | Yes | Yes | Yes | Yes | Yes | Low |
| Tung Gia Phan | Overall | Human | Homo sapiens | Viral Prevalence | Unclear | Unclear | No | No | No | Yes | Yes | No | Yes | High |
| Vivaldo Gomes da Costa | Test type | Human | Homo sapiens | Seroprevalence | Unclear | Yes | No | Yes | Yes | Yes | Yes | No | Yes | High |
| Vivaldo Gomes da Costa | Test type | Human | Homo sapiens | Seroprevalence | Unclear | Yes | No | Yes | Yes | Yes | Yes | No | Yes | High |
| Ana Carolina Bernardes Terzian | Overall | Human | Homo sapiens | Viral Prevalence | Yes | Unclear | Yes | No | Yes | Yes | Yes | Yes | Yes | Moderate |
| Liliana Sanchez-Lerma | Overall | Human | Homo sapiens | Viral Prevalence | No | No | Yes | Yes | Yes | Yes | Yes | No | Yes | Moderate |
| Juana del Valle-Mendoza | Gender | Human | Homo sapiens | Viral Prevalence | Yes | Yes | Yes | No | Yes | Yes | Yes | Yes | Yes | Moderate |
| Juana del Valle-Mendoza | Gender | Human | Homo sapiens | Viral Prevalence | Yes | Yes | Yes | No | Yes | Yes | Yes | Yes | Yes | Moderate |
| Douglas Watts | Test type | Human | Homo sapiens | Seroprevalence | Yes | Unclear | No | No | No | Yes | Yes | No | Yes | High |
| Douglas Watts | Test type | Human | Homo sapiens | Seroprevalence | Yes | Unclear | No | No | No | Yes | Yes | No | Yes | High |
| Ana Cecilia Ribeiro Cruz | Test type | Human | Homo sapiens | Seroprevalence | Yes | Unclear | Yes | Yes | No | Yes | Yes | Yes | Yes | Low |
| Ana Cecilia Ribeiro Cruz | Test type | Human | Homo sapiens | Seroprevalence | Yes | Unclear | Yes | Yes | No | Yes | Yes | Yes | Yes | Low |
| Laura Tauro | Overall | Insect | Culex quinquefasciatus | Viral Prevalence | Yes | Yes | Yes | No | No | Yes | Yes | Yes | Yes | Moderate |
| Laura Tauro | Overall | Insect | Aedes aegypti | Viral Prevalence | Yes | Yes | Yes | No | No | Yes | Yes | Yes | Yes | Moderate |
| F. P. Pinheiro | Species | Insect | Culicoides paraensis | Viral Prevalence | Yes | Unclear | No | Yes | No | Yes | Yes | No | Yes | Moderate |
| Belgath Fernandes Cardoso | Species | Insect | Culex quinquefasciatus | Viral Prevalence | Yes | Unclear | Yes | Yes | No | Yes | Yes | Yes | Yes | Low |
| Jordam Pereira-Silva | Overall | Insect | Multiple | Viral Prevalence | Yes | No | Yes | Yes | Yes | Yes | Yes | Yes | Yes | Low |
| Luiz Henrique Maciel Feitoza | Geography | Insect | Culicoides paraensis | Viral Prevalence | Yes | Unclear | No | No | Yes | Yes | Yes | No | Yes | High |
| Luiz Henrique Maciel Feitoza | Geography | Insect | Culicoides paraensis | Viral Prevalence | Yes | Unclear | No | No | Yes | Yes | Yes | No | Yes | High |
| Luiz Henrique Maciel Feitoza | Geography | Insect | Culicoides paraensis | Viral Prevalence | Yes | Unclear | No | Yes | Yes | Yes | Yes | No | Yes | Moderate |
| Raquel da Silva Ferreira | Species | Insect | Culex quinquefasciatus | Viral Prevalence | Yes | No | Yes | Yes | No | Yes | Yes | Yes | Yes | Low |
| Raquel da Silva Ferreira | Species | Insect | Aedes aegypti | Viral Prevalence | Yes | No | Yes | No | No | Yes | Yes | Yes | Yes | Moderate |
| Raquel da Silva Ferreira | Species | Insect | Culex Spp. | Viral Prevalence | Yes | No | Yes | No | No | Yes | Yes | Yes | Yes | Moderate |
| Raquel da Silva Ferreira | Species | Insect | Psorophora albigenu | Viral Prevalence | Yes | No | Yes | No | No | Yes | Yes | Yes | Yes | Moderate |
| Carlos Borborema | Species | Insect | Culicoides paraensis | Seroprevalence | Unclear | Unclear | Yes | Yes | No | Yes | Yes | No | Yes | High |
| Carlos Borborema | Species | Insect | Culex quinquefasciatus | Seroprevalence | Unclear | Unclear | Yes | No | No | Yes | Yes | No | Yes | High |
| Carlos Borborema | Species | Insect | Culex quinquefasciatus | Seroprevalence | Unclear | Unclear | Yes | No | No | Yes | Yes | No | Yes | High |
| Carlos Borborema | Species | Insect | Culicoides paraensis | Seroprevalence | Unclear | Unclear | Yes | No | No | Yes | Yes | No | Yes | High |
| Carlos Borborema | Species | Insect | Culex quinquefasciatus | Seroprevalence | Unclear | Unclear | Yes | No | No | Yes | Yes | No | Yes | High |
| Pedro Fernando da Costa Vasconcelos | Age | Insect | Culicoides paraensis | Seroprevalence | Unclear | No | Yes | No | Yes | Yes | Yes | No | Yes | High |
| Pedro Fernando da Costa Vasconcelos | Age | Insect | Anopheles nuneztovari | Seroprevalence | Unclear | No | Yes | No | Yes | Yes | Yes | No | Yes | High |
| Pedro Fernando da Costa Vasconcelos | Age | Insect | Anopheles triannulatus | Seroprevalence | Unclear | No | Yes | No | Yes | Yes | Yes | No | Yes | High |
| Pedro Fernando da Costa Vasconcelos | Age | Insect | Aedes scapularis | Seroprevalence | Unclear | No | Yes | No | Yes | Yes | Yes | No | Yes | High |
| Pedro Fernando da Costa Vasconcelos | Age | Insect | Psorophora cingulata | Seroprevalence | Unclear | No | Yes | No | Yes | Yes | Yes | No | Yes | High |
| Pedro Fernando da Costa Vasconcelos | Age | Insect | Psorophora ferox | Seroprevalence | Unclear | No | Yes | No | Yes | Yes | Yes | No | Yes | High |
| Pedro Fernando da Costa Vasconcelos | Age | Insect | Culex Spp. | Seroprevalence | Unclear | No | Yes | No | Yes | Yes | Yes | No | Yes | High |
| Pedro Fernando da Costa Vasconcelos | Age | Insect | Culex corniger | Seroprevalence | Unclear | No | Yes | No | Yes | Yes | Yes | No | Yes | High |
| Pedro Fernando da Costa Vasconcelos | Age | Insect | Culex coronator | Seroprevalence | Unclear | No | Yes | No | Yes | Yes | Yes | No | Yes | High |
| Pedro Fernando da Costa Vasconcelos | Age | Insect | Culex declarator | Seroprevalence | Unclear | No | Yes | No | Yes | Yes | Yes | No | Yes | High |
| Pedro Fernando da Costa Vasconcelos | Age | Insect | Culex quinquefasciatus | Seroprevalence | Unclear | No | Yes | No | Yes | Yes | Yes | No | Yes | High |
| Pedro Fernando da Costa Vasconcelos | Age | Insect | Mansonia Sp. | Seroprevalence | Unclear | No | Yes | No | Yes | Yes | Yes | No | Yes | High |
| Pedro Fernando da Costa Vasconcelos | Age | Insect | Limatus Sp | Seroprevalence | Unclear | No | Yes | No | Yes | Yes | Yes | No | Yes | High |
| Pedro Fernando da Costa Vasconcelos | Age | Insect | Sabethes glaucodaemon | Seroprevalence | Unclear | No | Yes | No | Yes | Yes | Yes | No | Yes | High |
| Pedro Fernando da Costa Vasconcelos | Age | Insect | Wyeomiya Sp. | Seroprevalence | Unclear | No | Yes | No | Yes | Yes | Yes | No | Yes | High |
| Pedro Fernando da Costa Vasconcelos | Age | Insect | Uranotaenia Sp | Seroprevalence | Unclear | No | Yes | No | Yes | Yes | Yes | No | Yes | High |
| Pedro Fernando da Costa Vasconcelos | Age | Insect | Culex Carrolia Sp | Seroprevalence | Unclear | No | Yes | No | Yes | Yes | Yes | No | Yes | High |
| Helver Dias | Species | Insect | Multiple | Viral Prevalence | Yes | No | Yes | Yes | Yes | Yes | Yes | Yes | Yes | Low |
| Helver Dias | Species | Insect | Wyeomiya Sp. | Viral Prevalence | Yes | No | Yes | Yes | Yes | Yes | Yes | Yes | Yes | Low |
| Helver Dias | Species | Insect | Aedes aegypti | Viral Prevalence | Yes | No | Yes | Yes | Yes | Yes | Yes | Yes | Yes | Low |
| Helver Dias | Species | Insect | Psorophora dimidiata | Viral Prevalence | Yes | No | Yes | No | Yes | Yes | Yes | Yes | Yes | Moderate |
| Helver Dias | Species | Insect | Culex nigripalpus | Viral Prevalence | Yes | No | Yes | Yes | Yes | Yes | Yes | Yes | Yes | Low |
| Helver Dias | Species | Insect | Psorophora albigenu | Viral Prevalence | Yes | No | Yes | Yes | Yes | Yes | Yes | Yes | Yes | Low |
| Helver Dias | Species | Insect | Aedes scapularis | Viral Prevalence | Yes | No | Yes | No | Yes | Yes | Yes | Yes | Yes | Moderate |
| Helver Dias | Species | Insect | Aedes albopictus | Viral Prevalence | Yes | No | Yes | No | Yes | Yes | Yes | Yes | Yes | Moderate |
| Helver Dias | Species | Insect | Psorophora Spp. | Viral Prevalence | Yes | No | Yes | No | Yes | Yes | Yes | Yes | Yes | Moderate |
| Helver Dias | Species | Insect | Culex quinquefasciatus | Viral Prevalence | Yes | No | Yes | No | Yes | Yes | Yes | Yes | Yes | Moderate |
| Helver Dias | Species | Insect | Aedes Spp. | Viral Prevalence | Yes | No | Yes | No | Yes | Yes | Yes | Yes | Yes | Moderate |
| Helver Dias | Species | Insect | Haemagogus janthinomys | Viral Prevalence | Yes | No | Yes | No | Yes | Yes | Yes | Yes | Yes | Moderate |
| Helver Dias | Species | Insect | Psorophora cingulata | Viral Prevalence | Yes | No | Yes | No | Yes | Yes | Yes | Yes | Yes | Moderate |
| Helver Dias | Species | Insect | Psorophora cilipes | Viral Prevalence | Yes | No | Yes | No | Yes | Yes | Yes | Yes | Yes | Moderate |
| Helver Dias | Species | Insect | Mansonia Sp. | Viral Prevalence | Yes | No | Yes | No | Yes | Yes | Yes | Yes | Yes | Moderate |
| Helver Dias | Species | Insect | Psorophora lanei | Viral Prevalence | Yes | No | Yes | No | Yes | Yes | Yes | Yes | Yes | Moderate |
| Helver Dias | Species | Insect | Haemagogus leucocelaenus | Viral Prevalence | Yes | No | Yes | No | Yes | Yes | Yes | Yes | Yes | Moderate |
| Helver Dias | Species | Insect | Sabethes Spp. | Viral Prevalence | Yes | No | Yes | No | Yes | Yes | Yes | Yes | Yes | Moderate |
| Helver Dias | Species | Insect | Haemagogus Sp. | Viral Prevalence | Yes | No | Yes | No | Yes | Yes | Yes | Yes | Yes | Moderate |
| Helver Dias | Species | Insect | Culex Spp. | Viral Prevalence | Yes | No | Yes | Yes | Yes | Yes | Yes | Yes | Yes | Low |
| Helver Dias | Species | Insect | Wyeomiya Sp. | Viral Prevalence | Yes | No | Yes | No | Yes | Yes | Yes | Yes | Yes | Moderate |
| Helver Dias | Species | Insect | Aedes aegypti | Viral Prevalence | Yes | No | Yes | No | Yes | Yes | Yes | Yes | Yes | Moderate |
| Helver Dias | Species | Insect | Psorophora dimidiata | Viral Prevalence | Yes | No | Yes | Yes | Yes | Yes | Yes | Yes | Yes | Low |
| Helver Dias | Species | Insect | Psorophora albigenu | Viral Prevalence | Yes | No | Yes | No | Yes | Yes | Yes | Yes | Yes | Moderate |
| Helver Dias | Species | Insect | Aedes scapularis | Viral Prevalence | Yes | No | Yes | No | Yes | Yes | Yes | Yes | Yes | Moderate |
| Helver Dias | Species | Insect | Aedes albopictus | Viral Prevalence | Yes | No | Yes | No | Yes | Yes | Yes | Yes | Yes | Moderate |
| Helver Dias | Species | Insect | Psorophora Spp. | Viral Prevalence | Yes | No | Yes | No | Yes | Yes | Yes | Yes | Yes | Moderate |
| Helver Dias | Species | Insect | Aedes Spp. | Viral Prevalence | Yes | No | Yes | No | Yes | Yes | Yes | Yes | Yes | Moderate |
| Helver Dias | Species | Insect | Haemagogus janthinomys | Viral Prevalence | Yes | No | Yes | No | Yes | Yes | Yes | Yes | Yes | Moderate |
| Helver Dias | Species | Insect | Psorophora cingulata | Viral Prevalence | Yes | No | Yes | No | Yes | Yes | Yes | Yes | Yes | Moderate |
| Helver Dias | Species | Insect | Psorophora cilipes | Viral Prevalence | Yes | No | Yes | No | Yes | Yes | Yes | Yes | Yes | Moderate |
| Helver Dias | Species | Insect | Mansonia Sp. | Viral Prevalence | Yes | No | Yes | No | Yes | Yes | Yes | Yes | Yes | Moderate |
| Helver Dias | Species | Insect | Psorophora lanei | Viral Prevalence | Yes | No | Yes | No | Yes | Yes | Yes | Yes | Yes | Moderate |
| Helver Dias | Species | Insect | Haemagogus leucocelaenus | Viral Prevalence | Yes | No | Yes | No | Yes | Yes | Yes | Yes | Yes | Moderate |
| Helver Dias | Species | Insect | Sabethes Spp. | Viral Prevalence | Yes | No | Yes | No | Yes | Yes | Yes | Yes | Yes | Moderate |
| Helver Dias | Species | Insect | Culex Spp. | Viral Prevalence | Yes | No | Yes | Yes | Yes | Yes | Yes | Yes | Yes | Low |
| Diego Michel Fernandes da Silva | Overall | Insect | Aedes aegypti | Viral Prevalence | Unclear | No | No | Yes | No | Yes | Yes | No | Yes | High |
| F. P. Pinheiro | Species | Non-human animal | Multiple | Seroprevalence | Yes | Unclear | No | Yes | No | Yes | Yes | No | Yes | Moderate |
| F. P. Pinheiro | Species | Non-human animal | Multiple | Seroprevalence | Yes | Unclear | No | No | No | Yes | Yes | No | Yes | High |
| F. P. Pinheiro | Species | Non-human animal | Multiple | Seroprevalence | Yes | Unclear | No | Yes | No | Yes | Yes | No | Yes | Moderate |
| F. P. Pinheiro | Species | Non-human animal | Multiple | Seroprevalence | Yes | Unclear | No | Yes | No | Yes | Yes | No | Yes | Moderate |
| Paulo Mira Batista | Gender | Non-human animal | Non-human primates | Seroprevalence | Yes | No | Yes | No | Yes | Yes | Yes | Yes | Yes | Moderate |
| Paulo Mira Batista | Gender | Non-human animal | Non-human primates | Seroprevalence | Yes | No | Yes | No | Yes | Yes | Yes | Yes | Yes | Moderate |
| Paulo Mira Batista | Age | Non-human animal | Non-human primates | Seroprevalence | Yes | No | Yes | No | Yes | Yes | Yes | Yes | Yes | Moderate |
| Paulo Mira Batista | Age | Non-human animal | Non-human primates | Seroprevalence | Yes | No | Yes | No | Yes | Yes | Yes | Yes | Yes | Moderate |
| Paulo Mira Batista | Geography | Non-human animal | Non-human primates | Seroprevalence | Yes | No | Yes | No | Yes | Yes | Yes | Yes | Yes | Moderate |
| Paulo Mira Batista | Geography | Non-human animal | Non-human primates | Seroprevalence | Yes | No | Yes | No | Yes | Yes | Yes | Yes | Yes | Moderate |
| Paulo Mira Batista | Geography | Non-human animal | Non-human primates | Seroprevalence | Yes | No | Yes | No | Yes | Yes | Yes | Yes | Yes | Moderate |
| Paulo Mira Batista | Geography | Non-human animal | Non-human primates | Seroprevalence | Yes | No | Yes | No | Yes | Yes | Yes | Yes | Yes | Moderate |
| Paulo Mira Batista | Geography | Non-human animal | Non-human primates | Seroprevalence | Yes | No | Yes | No | Yes | Yes | Yes | Yes | Yes | Moderate |
| Helver Gonçalves Dias | Species | Non-human animal | Bos indicus/taurus | Seroprevalence | Yes | Yes | Yes | No | Yes | Yes | Yes | Yes | Yes | Moderate |
| Helver Gonçalves Dias | Species | Non-human animal | Equus ferus caballus | Seroprevalence | Yes | Yes | Yes | No | Yes | Yes | Yes | Yes | Yes | Moderate |
| Helver Gonçalves Dias | Species | Non-human animal | Canis lupus familiaris | Seroprevalence | Yes | Yes | Yes | No | Yes | Yes | Yes | Yes | Yes | Moderate |
| Michael J. Turell | Overall | Non-human animal | Aotus nancymaae | Seroprevalence | No | Unclear | Yes | No | No | Yes | Yes | No | Yes | High |
| Plautino O. Laroque | Species | Non-human animal | Cebus flavius | Seroprevalence | Yes | Yes | Yes | No | No | Yes | Yes | Yes | Yes | Moderate |
| Plautino O. Laroque | Species | Non-human animal | Cebus libidinosus | Seroprevalence | Yes | Yes | Yes | Yes | Yes | Yes | Yes | Yes | Yes | Low |
| Alex Pauvolid-Correa | Species | Non-human animal | Equine species | Seroprevalence | Yes | Unclear | No | Yes | No | Yes | Yes | No | Yes | Moderate |
| Alex Pauvolid-Correa | Species | Non-human animal | Sheep species | Seroprevalence | Yes | Unclear | No | Yes | No | Yes | Yes | No | Yes | Moderate |
| Alex Pauvolid-Correa | Species | Non-human animal | Caiman species | Seroprevalence | Yes | Unclear | No | No | No | Yes | Yes | No | Yes | High |
| Helver Dias | Species | Non-human animal | Bos indicus/taurus | Viral Prevalence | Yes | No | Yes | No | Yes | Yes | Yes | Yes | Yes | Moderate |
| Helver Dias | Species | Non-human animal | Canis lupus familiaris | Viral Prevalence | Yes | No | Yes | No | Yes | Yes | Yes | Yes | Yes | Moderate |
| Helver Dias | Species | Non-human animal | Equus ferus caballus | Viral Prevalence | Yes | No | Yes | No | Yes | Yes | Yes | Yes | Yes | Moderate |
| Helver Dias | Species | Non-human animal | Felis silvestris catus | Viral Prevalence | Yes | No | Yes | No | Yes | Yes | Yes | Yes | Yes | Moderate |
| Helver Dias | Species | Non-human animal | Nasua nasua | Viral Prevalence | Yes | No | Yes | No | Yes | Yes | Yes | Yes | Yes | Moderate |
| Helver Dias | Species | Non-human animal | Didelphis albiventris | Viral Prevalence | Yes | No | Yes | No | Yes | Yes | Yes | Yes | Yes | Moderate |
| Helver Dias | Species | Non-human animal | Mico melanurus | Viral Prevalence | Yes | No | Yes | No | Yes | Yes | Yes | Yes | Yes | Moderate |
| Helver Dias | Species | Non-human animal | Sapajus apella | Viral Prevalence | Yes | No | Yes | No | Yes | Yes | Yes | Yes | Yes | Moderate |
| Helver Dias | Species | Non-human animal | Didelphis aurita | Viral Prevalence | Yes | No | Yes | No | Yes | Yes | Yes | Yes | Yes | Moderate |
| Helver Dias | Species | Non-human animal | Aotus lemurinus | Viral Prevalence | Yes | No | Yes | No | Yes | Yes | Yes | Yes | Yes | Moderate |
| Helver Dias | Species | Non-human animal | Ateles marginatus | Viral Prevalence | Yes | No | Yes | No | Yes | Yes | Yes | Yes | Yes | Moderate |
| Helver Dias | Species | Non-human animal | Bos indicus/taurus | Viral Prevalence | Yes | No | Yes | Yes | Yes | Yes | Yes | Yes | Yes | Low |
| Helver Dias | Species | Non-human animal | Canis lupus familiaris | Viral Prevalence | Yes | No | Yes | Yes | Yes | Yes | Yes | Yes | Yes | Low |
| Helver Dias | Species | Non-human animal | Equus ferus caballus | Viral Prevalence | Yes | No | Yes | No | Yes | Yes | Yes | Yes | Yes | Moderate |
| Helver Dias | Species | Non-human animal | Felis silvestris catus | Viral Prevalence | Yes | No | Yes | No | Yes | Yes | Yes | Yes | Yes | Moderate |
| Helver Dias | Species | Non-human animal | Nasua nasua | Viral Prevalence | Yes | No | Yes | No | Yes | Yes | Yes | Yes | Yes | Moderate |
| Helver Dias | Species | Non-human animal | Didelphis albiventris | Viral Prevalence | Yes | No | Yes | No | Yes | Yes | Yes | Yes | Yes | Moderate |
| Helver Dias | Species | Non-human animal | Sapajus cay | Viral Prevalence | Yes | No | Yes | No | Yes | Yes | Yes | Yes | Yes | Moderate |
| Helver Dias | Species | Non-human animal | Callithrix jacchus | Viral Prevalence | Yes | No | Yes | No | Yes | Yes | Yes | Yes | Yes | Moderate |
| Helver Dias | Species | Non-human animal | Alouatta caraya | Viral Prevalence | Yes | No | Yes | No | Yes | Yes | Yes | Yes | Yes | Moderate |
| Paulo Mira Batista | Overall | Non-human animal | Multiple | Seroprevalence | No | Unclear | No | No | No | Yes | Yes | No | Yes | High |
| Scott Medlin | Species | Non-human animal | Bradypus variegatus | Seroprevalence | Unclear | Unclear | Yes | No | No | Yes | Yes | No | Yes | High |
| Scott Medlin | Species | Non-human animal | Choloepus hoffmanni | Seroprevalence | Unclear | Unclear | Yes | No | No | Yes | Yes | No | Yes | High |
| Ana Cecilia Ribeiro Cruz | Species | Non-human animal | Phlegopsis n. pareensis | Seroprevalence | Yes | Unclear | Yes | No | No | Yes | Yes | Yes | Yes | Moderate |
| Ana Cecilia Ribeiro Cruz | Species | Non-human animal | Geotrygon montana | Seroprevalence | Yes | Unclear | Yes | No | No | Yes | Yes | Yes | Yes | Moderate |
| Ana Cecilia Ribeiro Cruz | Species | Non-human animal | Thamnophilus aethiops | Seroprevalence | Yes | Unclear | Yes | No | No | Yes | Yes | Yes | Yes | Moderate |
| Ana Cecilia Ribeiro Cruz | Species | Non-human animal | Conopophage roberti | Seroprevalence | Yes | Unclear | Yes | No | No | Yes | Yes | Yes | Yes | Moderate |
| Ana Cecilia Ribeiro Cruz | Species | Non-human animal | Schiffornis tudinus | Seroprevalence | Yes | Unclear | Yes | No | No | Yes | Yes | Yes | Yes | Moderate |
| Ana Cecilia Ribeiro Cruz | Species | Non-human animal | Xiphorhynchus ocellatus | Seroprevalence | Yes | Unclear | Yes | No | No | Yes | Yes | Yes | Yes | Moderate |
| Ana Cecilia Ribeiro Cruz | Species | Non-human animal | Myrmothera companisona | Seroprevalence | Yes | Unclear | Yes | No | No | Yes | Yes | Yes | Yes | Moderate |
| Ana Cecilia Ribeiro Cruz | Species | Non-human animal | Proechimis guianensis | Seroprevalence | Yes | Unclear | Yes | No | No | Yes | Yes | Yes | Yes | Moderate |
| Ana Cecilia Ribeiro Cruz | Species | Non-human animal | Didelphis marsupialis | Seroprevalence | Yes | Unclear | Yes | No | No | Yes | Yes | Yes | Yes | Moderate |

##

## Table D. Summary of species in vector studies

|  | **All studies** | **Studies included in meta-analysis (2000 and later)** |
| --- | --- | --- |
| **Study Species** | **N = 1251** | **N = 961** |
| *Aedes aegypti* | 5 (4.0%) | 5 (5.2%) |
| *Aedes albopictus* | 2 (1.6%) | 2 (2.1%) |
| *Aedes scapularis* | 3 (2.4%) | 2 (2.1%) |
| Unspecified *Aedes Spp.* | 2 (1.6%) | 2 (2.1%) |
| *Alouatta caraya* | 1 (0.8%) | 1 (1.0%) |
| *Anopheles nuneztovari* | 1 (0.8%) |  |
| *Anopheles triannulatus* | 1 (0.8%) |  |
| *Aotus lemurinus* | 1 (0.8%) | 1 (1.0%) |
| *Aotus nancymaae* | 1 (0.8%) | 1 (1.0%) |
| *Ateles marginatus* | 1 (0.8%) | 1 (1.0%) |
| *Bos indicus/taurus* | 3 (2.4%) | 3 (3.1%) |
| *Bradypus variegatus* | 1 (0.8%) | 1 (1.0%) |
| Unspecified caiman species | 1 (0.8%) | 1 (1.0%) |
| *Callithrix jacchus* | 1 (0.8%) | 1 (1.0%) |
| *Canis lupus familiaris* | 3 (2.4%) | 3 (3.1%) |
| *Cebus flavius* | 1 (0.8%) | 1 (1.0%) |
| *Cebus libidinosus* | 1 (0.8%) | 1 (1.0%) |
| *Choloepus hoffmanni* | 1 (0.8%) | 1 (1.0%) |
| *Conopophaga roberti* | 1 (0.8%) | 1 (1.0%) |
| Unspecified *Culex Carrolia Sp* | 1 (0.8%) |  |
| *Culex corniger* | 1 (0.8%) |  |
| *Culex coronator* | 1 (0.8%) |  |
| *Culex declarator* | 1 (0.8%) |  |
| *Culex nigripalpus* | 1 (0.8%) | 1 (1.0%) |
| *Culex quinquefasciatus* | 8 (6.4%) | 4 (4.2%) |
| Unspecified *Culex Spp.* | 4 (3.2%) | 4 (4.2%) |
| *Culicoides paraensis* | 7 (5.6%) | 3 (3.1%) |
| *Didelphis albiventris* | 2 (1.6%) | 2 (2.1%) |
| *Didelphis aurita* | 1 (0.8%) | 1 (1.0%) |
| *Didelphis marsupialis* | 1 (0.8%) | 1 (1.0%) |
| *Equus ferus caballus* | 4 (2.4%) | 4 (4.2%) |
| *Felis silvestris catus* | 2 (1.6%) | 2 (2.1%) |
| *Geotrygon montana* | 1 (0.8%) | 1 (1.0%) |
| *Haemagogus janthinomys* | 2 (1.6%) | 2 (2.1%) |
| *Haemagogus leucocelaenus* | 2 (1.6%) | 2 (2.1%) |
| Unspecified *Haemagogus Sp.* | 1 (0.8%) | 1 (1.0%) |
| Unspecified *Limatus Sp* | 1 (0.8%) |  |
| Unspecified *Mansonia Sp.* | 3 (2.4%) | 2 (2.1%) |
| *Mico melanurus* | 1 (0.8%) | 1 (1.0%) |
| *Myrmothera campanisona* | 1 (0.8%) | 1 (1.0%) |
| *Nasua nasua* | 2 (1.6%) | 2 (2.1%) |
| Unspecified non-human primates | 9 (7.2%) | 8 (8.3%) |
| *Phlegopsis n. pareensis* | 1 (0.8%) | 1 (1.0%) |
| *Proechimys guianensis* | 1 (0.8%) | 1 (1.0%) |
| *Psorophora albigenu* | 3 (2.4%) | 3 (3.1%) |
| *Psorophora cilipes* | 2 (1.6%) | 2 (2.1%) |
| *Psorophora cingulata* | 3 (2.4%) | 2 (2.1%) |
| *Psorophora dimidiata* | 2 (1.6%) | 2 (2.1%) |
| *Psorophora ferox* | 1 (0.8%) |  |
| *Psorophora lanei* | 2 (1.6%) | 2 (2.1%) |
| Unspecified *Psorophora Spp.* | 2 (1.6%) | 2 (2.1%) |
| *Sabethes glaucodaemon* | 1 (0.8%) |  |
| Unspecified *Sabethes Spp.* | 2 (1.6%) | 2 (2.1%) |
| *Sapajus apella* | 1 (0.8%) | 1 (1.0%) |
| *Sapajus cay* | 1 (0.8%) | 1 (1.0%) |
| *Schiffornis tudinus* | 1 (0.8%) | 1 (1.0%) |
| Unspecified sheep species | 1 (0.8%) | 1 (1.0%) |
| *Thamnophilus aethiops* | 1 (0.8%) | 1 (1.0%) |
| Unspecified *Uranotaenia Sp* | 1 (0.8%) |  |
| Unspecified *Wyeomyia Sp.* | 3 (2.4%) | 2 (2.1%) |
| *Xiphorhynchus ocellatus* | 1 (0.8%) | 1 (1.0%) |
| Unspecified insect | 7 (5.6%) | 1 (1.0%) |

## Fig A. Sensitivity Analysis: Reported pooled seroprevalence from studies using screening assays only, sampling participants with febrile illness or suspected of Oropouche infection in 2000 to 2024.

Seroprevalence is displayed as a proportion with 95% confidence intervals in square brackets.


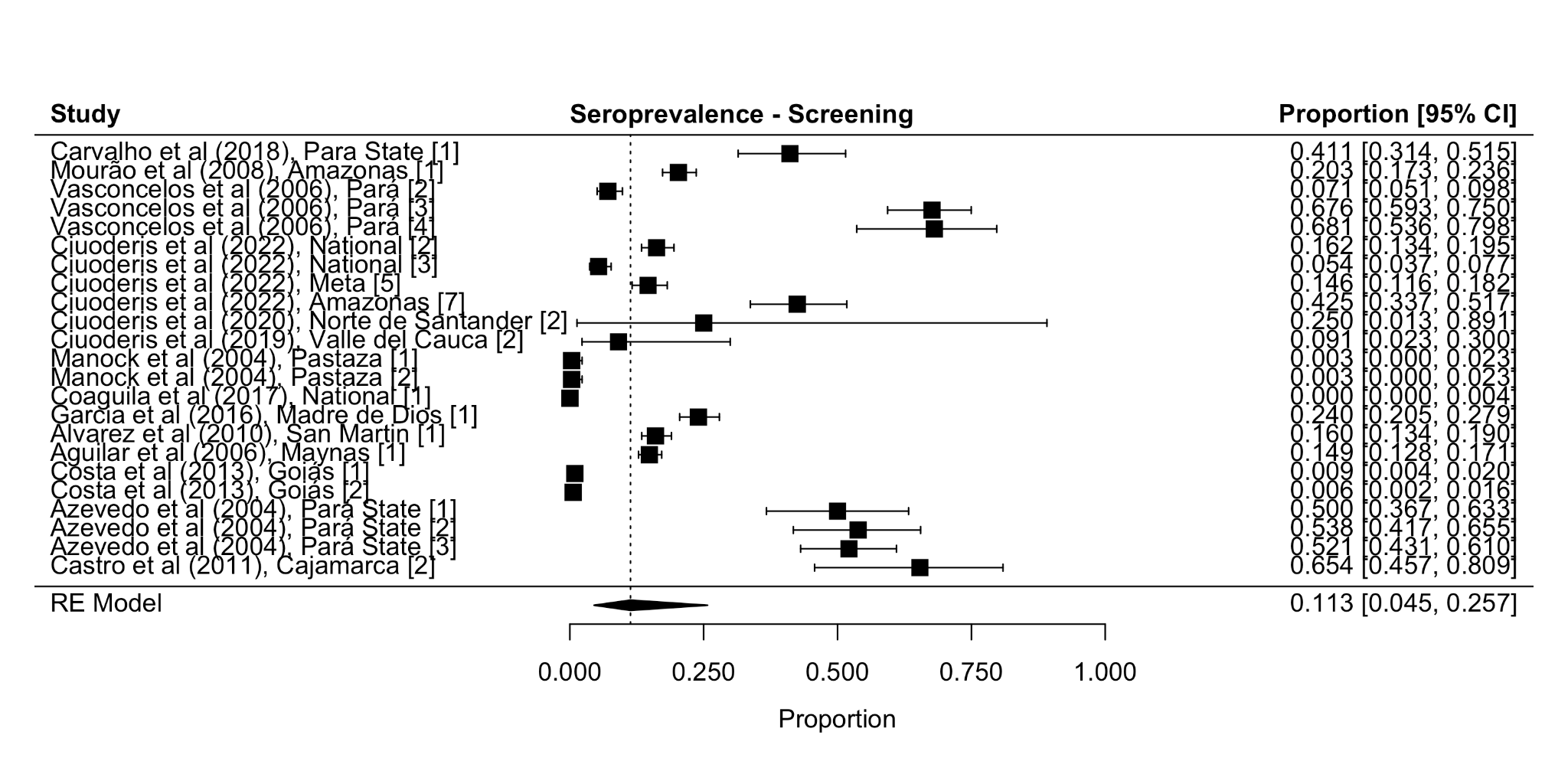


## Fig B. Sensitivity Analysis: Reported pooled seroprevalence from studies using neutralizing assays, sampling participants with febrile illness or suspected of Oropouche infection in 2000 to 2024.

Seroprevalence is displayed as a proportion with 95% confidence intervals in square brackets.


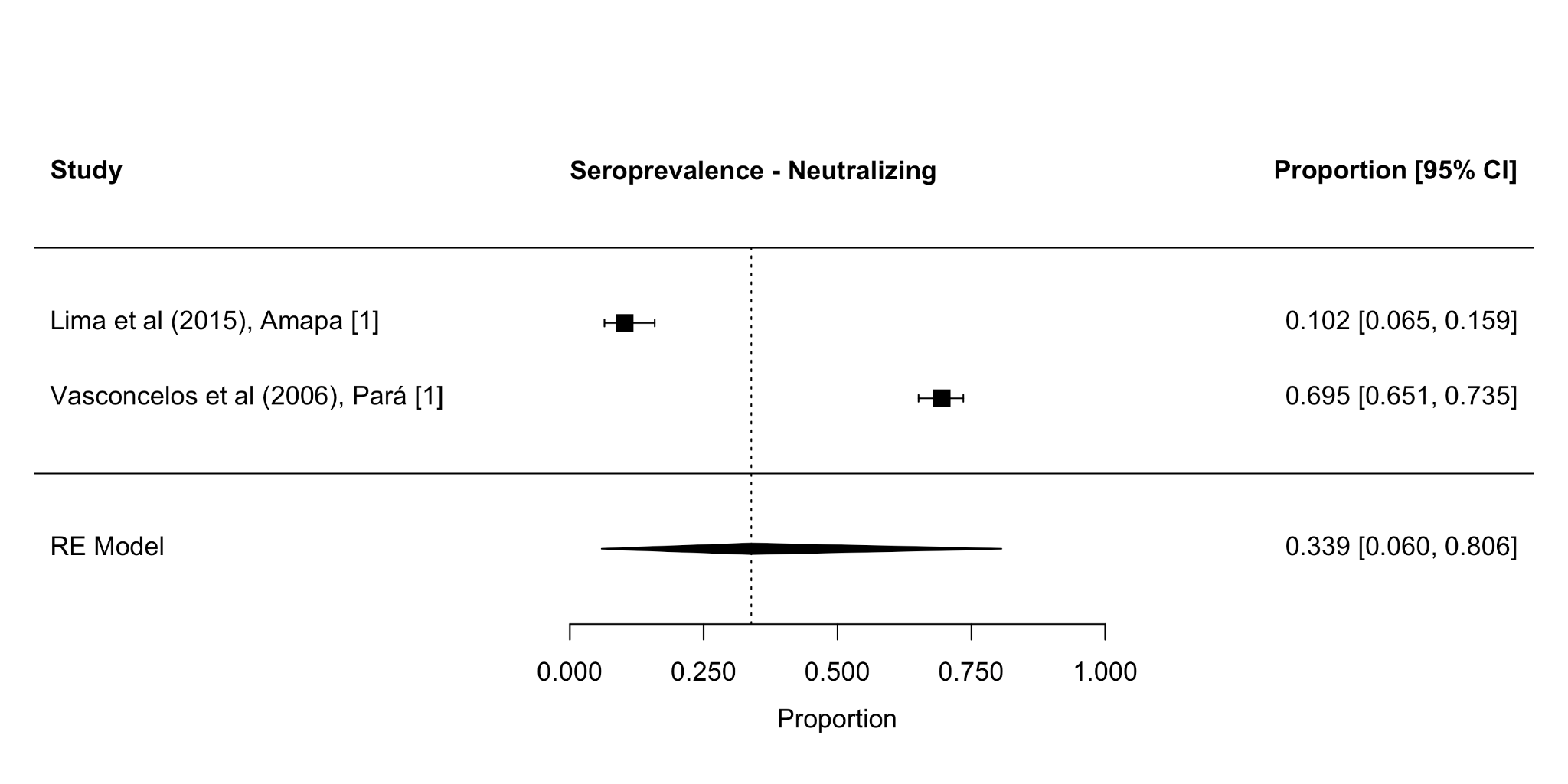


## Fig C. Sensitivity Analysis: Reported pooled seroprevalence from studies using screening assays only, sampling participants among asymptomatic general populations in 2000 to 2024.

Seroprevalence is displayed as a proportion with 95% confidence intervals in square brackets.


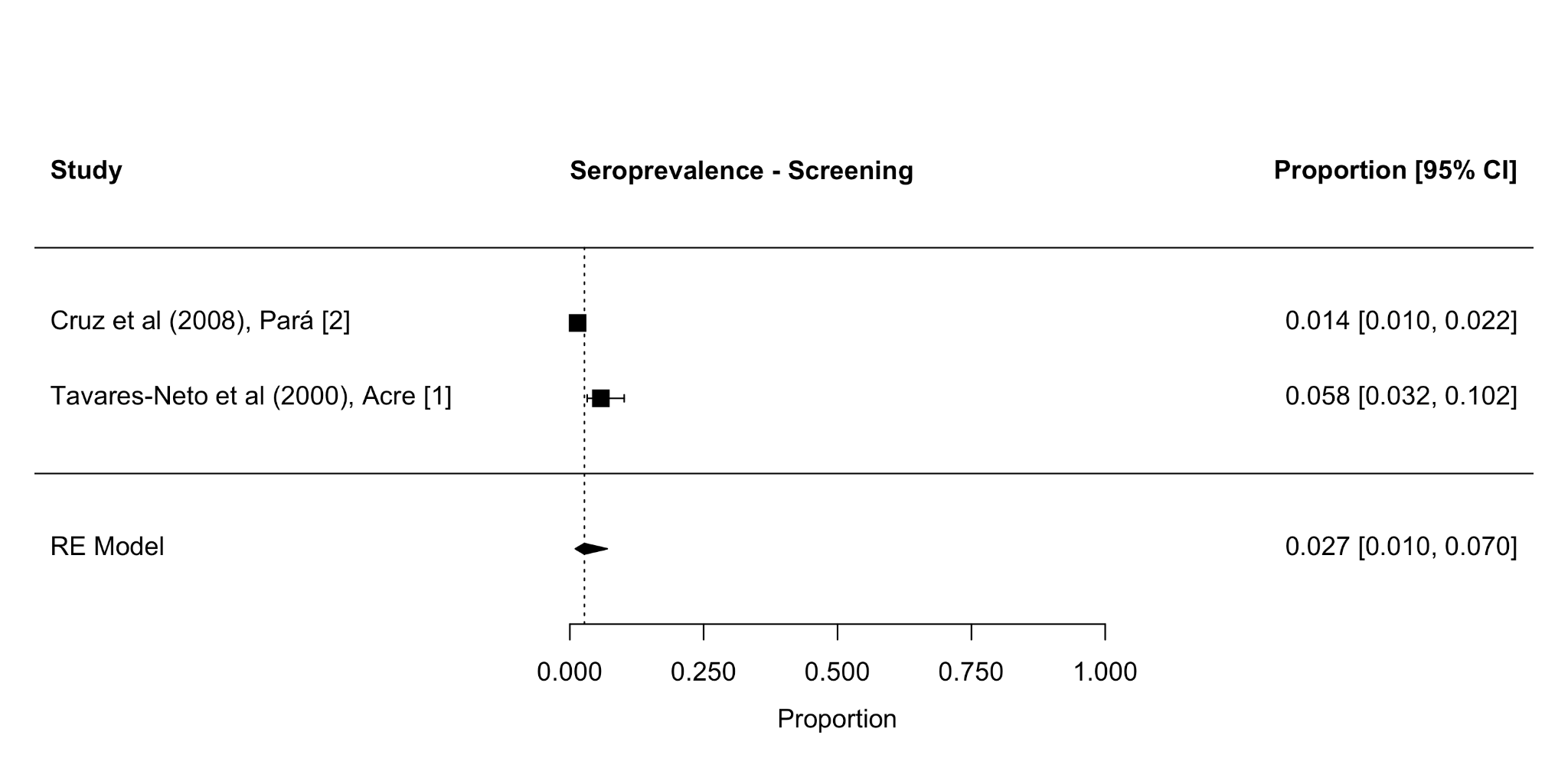


## Fig D. Sensitivity Analysis: Reported pooled seroprevalence from studies using neutralizing assays, sampling participants among asymptomatic general populations in 2000 to 2024.

Seroprevalence is displayed as a proportion with 95% confidence intervals in square brackets.


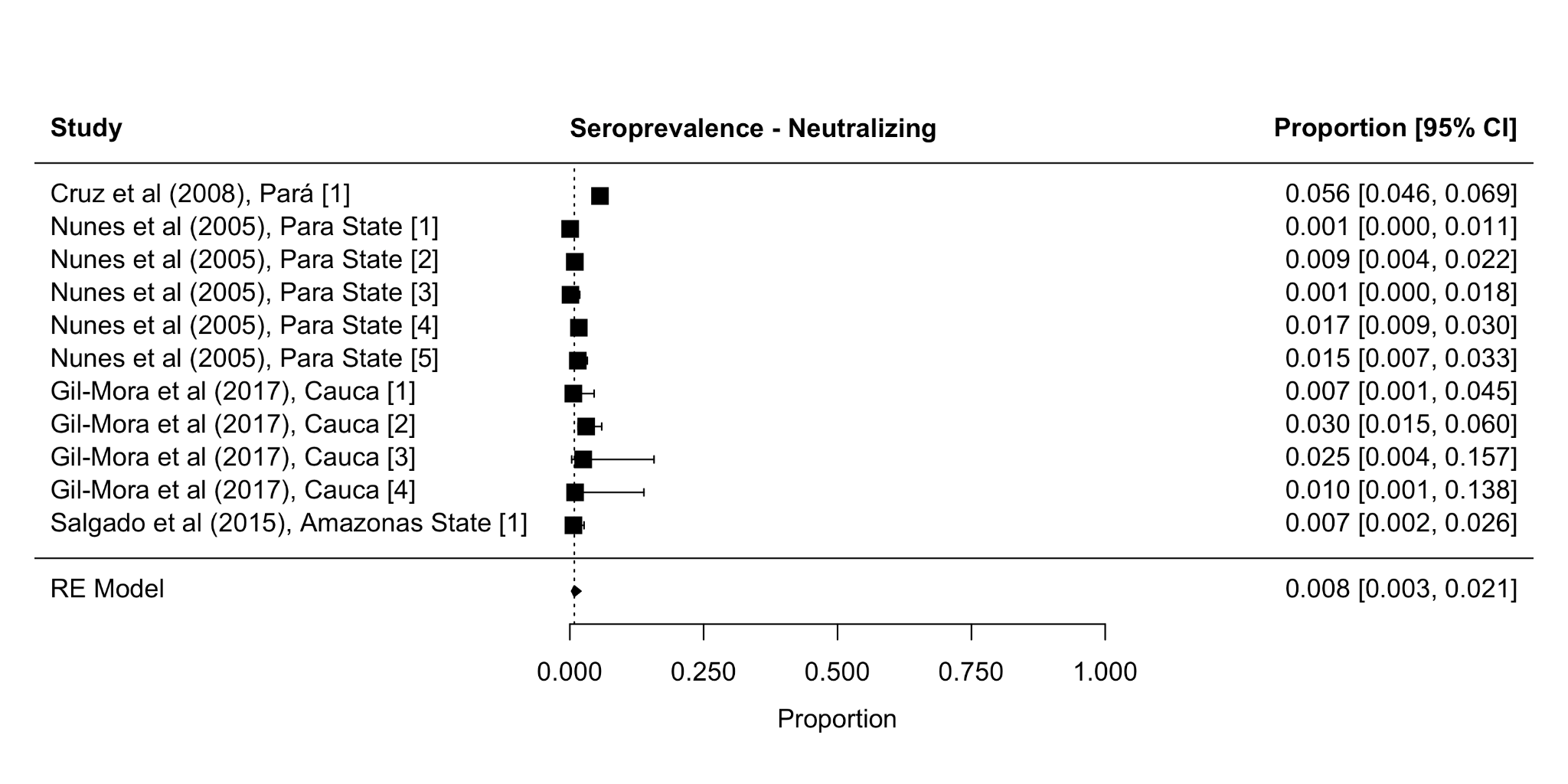


**References**

1. Pinheiro FP, Travassos Da Rosa APA, Travassos Da Rossa JFG. An outbreak of Oropouche virus disease in the vicinity of Santarem, Para, Brazil. Tropenmedizin und Parasitologie. 1976;27: 213–223.

2. LeDuc JW, Hoch AL, Pinheiro FP, Travassos da Rosa APA. Epidemic Oropouche virus disease in northern Brazil. 1981;15: 97–103.

3. Freitas RB, Pinheiro FP, Santos MAV, Travassos Da Rosa APDA, Travassos Da Rosa JFS, de Freitas EN. Epidemia de vírus Oropouche no leste do estado do Pará, 1979. Academia Brasileira de Ciências. 1982; 419–432.

4. Borborema CAT, Pinheiro FP, Albuquerque BC, da Rosa ACT, da Rosa JFST, Dourado HV. Primeiro registro de epidemias causadas pelo virus Oropouche no estado do Amazonas. Revista do Instituto de Medicina Tropical de Sao Paulo. 1982;24: 132–139.

5. Vasconcelos PFDC, Travassos Da Rosa JFS, Guerreiro SC, Dégallier N, Travassos Da Rosa ES, Travassos Da Rosa APDA. Primeiro registro de epidemias causadas pelo vírus Oropouche nos Estados do Maranhão e Goiás, Brasil. Revista do Instituto de Medicina Tropical de São Paulo. 1989;31: 271–278. doi:[10.1590/S0036-46651989000400011](https://doi.org/10.1590/S0036-46651989000400011)

6. Watts DM, Lavera V, Callahan J, Rossi C, Oberste SM, Roehrig JT, et al. Venezuelan equine encephalitis and Oropouche virus infections among Peruvian army troops in the Amazon region of Peru. The American Journal of Tropical Medicine and Hygiene. 1997;56: 661–667. doi:[10.4269/ajtmh.1997.56.661](https://doi.org/10.4269/ajtmh.1997.56.661)

7. Rosa APAT, Rodrigues SG, Nunes MRT, Magalhães MTF, Rosa JFST, Vasconcelos PFC. Epidemia de febre do Oropouche em Serra Pelada, município de Curionópolis, Pará, 1994. Revista da Sociedade Brasileira de Medicina Tropical. 1996;29: 537–541. doi:[10.1590/S0037-86821996000600002](https://doi.org/10.1590/S0037-86821996000600002)

8. Baisley KJ, Watts DM, Munstermann LE, Wilson ML. Epidemiology of endemic Oropouche virus transmission in upper Amazonian Peru. The American journal of tropical medicine and hygiene. 2001;59: 710–6.

9. Tavares-Neto J, Freitas-Carvalho J, Nunes MRT, Rocha G, Rodrigues SG, Damasceno E, et al. Pesquisa de anticorpos contra arbovírus e o vírus vacinal da febre amarela em uma amostra da população de Rio Branco, antes e três meses após a vacina 17D. Revista da Sociedade Brasileira de Medicina Tropical. 2004;37: 1–6.

10. Watts DM, Russell KL, Wooster MT, Sharp TW, Morrison AC, Kochel TJ, et al. Etiologies of Acute Undifferentiated Febrile Illnesses in and near Iquitos from 1993 to 1999 in the Amazon River Basin of Peru. The American journal of tropical medicine and hygiene. 2022;107: 1114–1128. doi:[10.4269/ajtmh.22-0259](https://doi.org/10.4269/ajtmh.22-0259)

11. Figueiredo RMPD, Thatcher BD, Lima MLD, Almeida TC, Alecrim WD, Guerra MVDF. Doenças exantemáticas e primeira epidemia de dengue ocorrida em Manaus, Amazonas, no período de 1998-1999. Revista da Sociedade Brasileira de Medicina Tropical. 2004;37: 476–479. doi:[10.1590/S0037-86822004000600009](https://doi.org/10.1590/S0037-86822004000600009)

12. Azevedo R do S da S, Nunes MRT, Chiang JO, Bensabath G, Vasconcelos HB, Pinto AY das N, et al. Reemergence of Oropouche Fever, Northern Brazil - Volume 13, Number 6—June 2007 - Emerging Infectious Diseases journal - CDC. doi:[10.3201/eid1306.061114](https://doi.org/10.3201/eid1306.061114)

13. Manock SR, Jacobsen KH, De Bravo NB, Russell KL, Negrete M, Olson JG, et al. Etiology of Acute Undifferentiated Febrile Illness in the Amazon Basin of Ecuador. The American Journal of Tropical Medicine and Hygiene. 2009;81: 146–151. doi:[10.4269/ajtmh.2009.81.146](https://doi.org/10.4269/ajtmh.2009.81.146)

14. Nunes MRT, Barbosa TFS, Casseb LMN, Nunes Neto JP, Segura N de O, Monteiro HA de O, et al. Eco-epidemiologia dos arbovírus na área de influência da rodovia Cuiabá-Santarém (BR 163), Estado do Pará, Brasil. Cadernos de Saúde Pública. 2009;25: 2583–2602. doi:[10.1590/S0102-311X2009001200006](https://doi.org/10.1590/S0102-311X2009001200006)

15. Vasconcelos HB, Azevedo RSS, Casseb SM, Nunes-Neto JP, Chiang JO, Cantuária PC, et al. Oropouche fever epidemic in Northern Brazil: Epidemiology and molecular characterization of isolates. Journal of Clinical Virology. 2009;44: 129–133. doi:[10.1016/j.jcv.2008.11.006](https://doi.org/10.1016/j.jcv.2008.11.006)

16. Terzian ACB, Bronzoni RV de M, Drumond BP, Silva-Nunes MD, da Silva NS, Ferreira MU, et al. Sporadic Oropouche Infection, Acre, Brazil. Emerging Infectious Diseases. 2009;15: 348–350. doi:[10.3201/eid1502.080401](https://doi.org/10.3201/eid1502.080401)

17. Aguilar PV, Barrett AD, Saeed MF, Watts DM, Russell K, Guevara C, et al. Iquitos Virus: A Novel Reassortant Orthobunyavirus Associated with Human Illness in Peru. Turell MJ, editor. PLoS Neglected Tropical Diseases. 2011;5: e1315. doi:[10.1371/journal.pntd.0001315](https://doi.org/10.1371/journal.pntd.0001315)

18. Forshey BM, Guevara C, Laguna-Torres VA, Cespedes M, Vargas J, Gianella A, et al. Arboviral Etiologies of Acute Febrile Illnesses in Western South America, 2000–2007. Halstead SB, editor. PLoS Neglected Tropical Diseases. 2010;4: e787. doi:[10.1371/journal.pntd.0000787](https://doi.org/10.1371/journal.pntd.0000787)

19. Cruz ACR, dos Prazeres A do SC, Gama EC, de Lima MF, Azevedo R do SS, Casseb LMN, et al. Vigilância sorológica para arbovírus em Juruti, Pará, Brasil. Cadernos de Saúde Pública. 2009;25: 2517–2523. doi:[10.1590/S0102-311X2009001100021](https://doi.org/10.1590/S0102-311X2009001100021)

20. Mourão MPG, Bastos MS, Gimaque JBL, Mota BR, Souza GS, Grimmer GHN, et al. Oropouche Fever Outbreak, Manaus, Brazil, 2007–2008 - Volume 15, Number 12—December 2009 - Emerging Infectious Diseases journal - CDC. 2009. doi:[10.3201/eid1512.090917](https://doi.org/10.3201/eid1512.090917)

21. Alvarez-Falconi PP, Ríos Ruiz BA. [Oropuche fever outbreak in Bagazan, San Martin, Peru: epidemiological evaluation, gastrointestinal and hemorrhagic manifestations]. Revista De Gastroenterologia Del Peru: Organo Oficial De La Sociedad De Gastroenterologia Del Peru. 2010;30: 334–340.

22. Martins V do CA, Bastos M de S, Ramasawmy R, de Figueiredo RP, Gimaque JBL, Braga WSM, et al. Clinical and Virological Descriptive Study in the 2011 Outbreak of Dengue in the Amazonas, Brazil. PLOS ONE. 2014;9: e100535. doi:[10.1371/journal.pone.0100535](https://doi.org/10.1371/journal.pone.0100535)

23. Castro S, Banda L, Cabellos D, Luna D, Muñoz J, Cley Condor Y. Brote de fiebre de Oropuche en dos localidades de la región Cajamarca, Perú, 2011 Rev. Peru. Epidemiol. (Online);17(3): 1-6, sept.-dic. 2013. Map, tab, graf LILACS LIPECS. Revista Peruana de Epidemiologia. 2013;17: 1–6.

24. Cardoso BF, Serra OP, Heinen LB da S, Zuchi N, de Souza VC, Naveca FG, et al. Detection of Oropouche virus segment S in patients and in*Culex* *Quinquefasciatus* in the state of Mato Grosso, Brazil. Memórias do Instituto Oswaldo Cruz. 2015;110: 745–754. doi:[10.1590/0074-02760150123](https://doi.org/10.1590/0074-02760150123)

25. Bastos MS, Lessa N, Naveca FG, Monte RL, Braga WS, Figueiredo LTM, et al. Detection of Herpesvirus, Enterovirus, and Arbovirus infection in patients with suspected central nervous system viral infection in the Western Brazilian Amazon. Journal of Medical Virology. 2014;86: 1522–1527. doi:[10.1002/jmv.23953](https://doi.org/10.1002/jmv.23953)

26. da Costa VG, de Rezende Féres VC, Saivish MV, de Lima Gimaque JB, Moreli ML. Silent emergence of Mayaro and Oropouche viruses in humans in Central Brazil. International Journal of Infectious Diseases. 2017;62: 84–85. doi:[10.1016/j.ijid.2017.07.016](https://doi.org/10.1016/j.ijid.2017.07.016)

27. Elbadry MA, Durães-Carvalho R, Blohm GM, Stephenson CJ, Loeb JC, White SK, et al. Orthobunyaviruses in the Caribbean: Melao and Oropouche virus infections in school children in Haiti in 2014. PLOS Neglected Tropical Diseases. 2021;15: e0009494. doi:[10.1371/journal.pntd.0009494](https://doi.org/10.1371/journal.pntd.0009494)

28. de Lima RC, Dias HG, de Souza TM, Familiar-Macedo D, Ribeiro ED, Corrêa VC, et al. Oropouche Virus Exposure in Febrile Patients during Chikungunya Virus Introduction in the State of Amapá, Amazon Region, Brazil. Pathogens (Basel, Switzerland). 2024;13. doi:[10.3390/pathogens13060469](https://doi.org/10.3390/pathogens13060469)

29. Naveca FG, Nascimento VA, Souza VC, de Figueiredo RMP. Human Orthobunyavirus Infections, Tefé, Amazonas, Brazil. PLOS Currents Outbreaks. 2018. doi:[10.1371/currents.outbreaks.7d65e5eb6ef75664da68905c5582f7f7](https://doi.org/10.1371/currents.outbreaks.7d65e5eb6ef75664da68905c5582f7f7)

30. Salgado BB, De Jesus Maués FC, Pereira RL, Chiang JO, De Oliveira Freitas MN, Ferreira MS, et al. Prevalence of arbovirus antibodies in young healthy adult population in Brazil. Parasites & Vectors. 2021;14: 403. doi:[10.1186/s13071-021-04901-4](https://doi.org/10.1186/s13071-021-04901-4)

31. García MP, Merino NS, Figueroa D, Marcelo A, V ET, Manrique C, et al. Detección de la circulación del virus Oropuche en la región Madre de Dios, Perú (diciembre 2015 - enero 2016). Revista Peruana de Medicina Experimental y Salud Pública. 2016; 380–381. doi:[10.17843/rpmesp.2016.332.2098](https://doi.org/10.17843/rpmesp.2016.332.2098)

32. Phan TG, del Valle Mendoza J, Sadeghi M, Altan E, Deng X, Delwart E. Sera of Peruvians with fever of unknown origins include viral nucleic acids from non-vertebrate hosts. Virus Genes. 2018;54: 33–40. doi:[10.1007/s11262-017-1514-3](https://doi.org/10.1007/s11262-017-1514-3)

33. Alva-Urcia C, Aguilar-Luis MA, Palomares-Reyes C, Silva-Caso W, Suarez-Ognio L, Weilg P, et al. Emerging and reemerging arboviruses: A new threat in Eastern Peru. PLOS ONE. 2017;12: e0187897. doi:[10.1371/journal.pone.0187897](https://doi.org/10.1371/journal.pone.0187897)

34. Dias HG, de Lima RC, Barbosa LS, de Souza TMA, Badolato-Correa J, Maia LMS, et al. Retrospective molecular investigation of Mayaro and Oropouche viruses at the human-animal interface in West-central Brazil, 2016–2018. PLOS ONE. 2022;17: e0277612. doi:[10.1371/journal.pone.0277612](https://doi.org/10.1371/journal.pone.0277612)

35. Nascimento VA do, Santos JHA, Monteiro DC da S, Pessoa KP, Cardoso AJL, Souza VC de, et al. Oropouche virus detection in saliva and urine. Memórias do Instituto Oswaldo Cruz. 2020;115: e190338. doi:[10.1590/0074-02760190338](https://doi.org/10.1590/0074-02760190338)

36. del Valle-Mendoza J, Vasquez-Achaya F, Aguilar-Luis MA, Martins-Luna J, Bazán-Mayra J, Zavaleta-Gavidia V, et al. Unidentified dengue serotypes in DENV positive samples and detection of other pathogens responsible for an acute febrile illness outbreak 2016 in Cajamarca, Peru. BMC Research Notes. 2020;13: 467. doi:[10.1186/s13104-020-05318-5](https://doi.org/10.1186/s13104-020-05318-5)

37. Durango-Chavez HV, Toro-Huamanchumo CJ, Silva-Caso W, Martins-Luna J, Aguilar-Luis MA, Valle-Mendoza J del, et al. Oropouche virus infection in patients with acute febrile syndrome: Is a predictive model based solely on signs and symptoms useful? PLOS ONE. 2022;17: e0270294. doi:[10.1371/journal.pone.0270294](https://doi.org/10.1371/journal.pone.0270294)

38. de Souza Costa MC, Siqueira Maia LM, Costa de Souza V, Gonzaga AM, Correa de Azevedo V, Ramos Martins L, et al. Arbovirus investigation in patients from Mato Grosso during Zika and Chikungunya virus introdution in Brazil, 2015–2016. Acta Tropica. 2019;190: 395–402. doi:[10.1016/j.actatropica.2018.12.019](https://doi.org/10.1016/j.actatropica.2018.12.019)

39. Silva-Caso W, Aguilar-Luis MA, Palomares-Reyes C, Mazulis F, Weilg C, del Valle LJ, et al. First outbreak of Oropouche Fever reported in a non-endemic western region of the Peruvian Amazon: Molecular diagnosis and clinical characteristics. International Journal of Infectious Diseases. 2019;83: 139–144. doi:[10.1016/j.ijid.2019.04.011](https://doi.org/10.1016/j.ijid.2019.04.011)

40. Silva-Caso W, Palomares-Reyes C, Aguilar-Luis MA, Sacramento-Meléndez J, Espejo-Evaristo B, Soto-Febres F, et al. Oropouche virus identification as an emerging etiological agent responsible for acute febrile disease in a eastern middle region of the Peruvian jungle. Sixty-sixth Annual Meeting. Baltimore, Maryland USA: American Society of Tropical Medicine and Hygiene; 2017. pp. 262–263.

41. Martins-Luna J, Mazulis F, Aguilar-Luis MA, Silva-Caso W, Weilg C, del Valle LJ, et al. OROV isolation in a northern region of Peru: First molecular Identification and clinical characteristics. American Journal of Tropical Medicine and Hygiene. 2018;99: 77–78.

42. Martins-Luna J, del Valle-Mendoza J, Silva-Caso W, Sandoval I, del Valle LJ, Palomares-Reyes C, et al. Oropouche infection a neglected arbovirus in patients with acute febrile illness from the Peruvian coast. BMC Research Notes. 2020;13: 67. doi:[10.1186/s13104-020-4937-1](https://doi.org/10.1186/s13104-020-4937-1)

43. del Valle-Mendoza J, Aguilar-Luis MA, Vasquez-Achaya F, Mayra JB, Zavaleta-Gavidia V, Cornejo-Pacherres D, et al. An outbreak of febrile syndromes in the north of Peru: Emerging and reemergin arboviruses. American Society of Tropical Medicine and Hygiene. 2017;97.

44. Saatkamp CJ, Rodrigues LRR, Pereira AMN, Coelho JA, Marques RGB, de Souza VC, et al. Mayaro virus detection in the western region of Pará state, Brazil. Revista da Sociedade Brasileira de Medicina Tropical. 2021;54: e0055–2020. doi:[10.1590/0037-8682-0055-2020](https://doi.org/10.1590/0037-8682-0055-2020)

45. Wise EL, Márquez S, Mellors J, Paz V, Atkinson B, Gutierrez B, et al. Oropouche virus cases identified in Ecuador using an optimised qRT-PCR informed by metagenomic sequencing. PLOS Neglected Tropical Diseases. 2020;14: e0007897. doi:[10.1371/journal.pntd.0007897](https://doi.org/10.1371/journal.pntd.0007897)

46. Coaguila M, Garcia M, Figueroa, Merino N, Marcelo A, Cobos M, et al. Re-emerging of Mayaro virus in areas with circulation of dengue virus in the Peruvian Amazon. American Society of Tropical Medicine and Hygiene. 2017;97: 2–3.

47. Gil-Mora J, Acevedo-Gutiérrez LY, Betancourt-Ruiz PL, Martínez-Diaz HC, Fernández D, Bopp NE, et al. Arbovirus Antibody Seroprevalence in the Human Population from Cauca, Colombia. 2022. doi:[10.4269/ajtmh.22-0120](https://doi.org/10.4269/ajtmh.22-0120)

48. Fonseca LM dos S, Carvalho RH, Bandeira AC, Sardi SI, Campos GS. Oropouche Virus Detection in Febrile Patients’ Saliva and Urine Samples in Salvador, Bahia, Brazil. Japanese Journal of Infectious Diseases. 2020;73: 164–165. doi:[10.7883/yoken.JJID.2019.296](https://doi.org/10.7883/yoken.JJID.2019.296)

49. Carvalho VL, Azevedo RSS, Carvalho VL, Azevedo RS, Henriques DF, Cruz ACR, et al. Arbovirus outbreak in a rural region of the Brazilian Amazon. Journal of Clinical Virology. 2022;150–151: 105155. doi:[10.1016/j.jcv.2022.105155](https://doi.org/10.1016/j.jcv.2022.105155)

50. Pavon JAR, Neves NA da S, Silva LCF, Azevedo FK de, Junior JAB de F, Nunes MRT, et al. Neurological infection by chikungunya and a triple Arbovirus co-infection in Mato Grosso, Central Western Brazil during 2019. Journal of Clinical Virology. 2022;146: 105056. doi:[10.1016/j.jcv.2021.105056](https://doi.org/10.1016/j.jcv.2021.105056)

51. Gonçalves Maciel LH, Vieira da Rocha Neto C, Ferreira Martins Y, de Azevedo Furtado F, Cunha Teixeira P, Oliveira Dias MY, et al. Prevalence of arboviruses and other infectious causes of skin rash in patients treated at a tertiary health unit in the Brazilian Amazon. PLOS Neglected Tropical Diseases. 2022;16: e0010727. doi:[10.1371/journal.pntd.0010727](https://doi.org/10.1371/journal.pntd.0010727)

52. Queiroz JA da S, Botelho-Souza LF, Nogueira-Lima FS, Rampazzo R de CP, Krieger MA, Zambenedetti MR, et al. Phylogenetic Characterization of Arboviruses in Patients Suffering from Acute Fever in Rondônia, Brazil. Viruses. 2020;12: 889. doi:[10.3390/v12080889](https://doi.org/10.3390/v12080889)

53. da Silva DMF, de Curcio JS, Moura YCD, Garcia-Zapata MTA, Annunciation CE, Lacerda E de PS. Rastreio do virus Oropouche no estado de Goias. Brazillian Journal of Infectious Diseases. 2022;26: 101736.

54. Gaillet M, Pichard C, Restrepo J, Lavergne A, Perez L, Enfissi A, et al. Outbreak of Oropouche Virus in French Guiana. Emerging Infectious Diseases. 2021;27: 2711–2714. doi:[10.3201/eid2710.204760](https://doi.org/10.3201/eid2710.204760)

55. Silva-Ramos CR, Gil-Mora J, Serna-Rivera CC, Martínez Díaz H-C, Restrepo-López N, Agudelo-Flórez P, et al. Etiological characterization of acute undifferentiated febrile illness in Apartadó and Villeta municipalities, Colombia, during COVID-19 pandemic. Le Infezioni in Medicina. 2023;31: 517–532. doi:[10.53854/liim-3104-11](https://doi.org/10.53854/liim-3104-11)

56. Ciuoderis KA, Berg MG, Perez LJ, Hadji A, Perez-Restrepo LS, Aristizabal LC, et al. Oropouche virus as an emerging cause of acute febrile illness in Colombia. Emerging Microbes & Infections. 2022;11: 2645–2657. doi:[10.1080/22221751.2022.2136536](https://doi.org/10.1080/22221751.2022.2136536)

57. Moreira HM, Sgorlon G, Queiroz JAS, Roca TP, Ribeiro J, Teixeira KS, et al. Outbreak of Oropouche virus in frontier regions in western Amazon. Microbiology Spectrum. 2024;12: e01629–23. doi:[10.1128/spectrum.01629-23](https://doi.org/10.1128/spectrum.01629-23)

58. Sánchez-Lerma L, Rojas-Gulloso A, Miranda J, Tique V, Patiño LH, Rodriguez D, et al. Unexpected arboviruses found in an epidemiological surveillance of acute tropical febrile syndrome in the department of Meta, Eastern Colombia. Journal of Infection and Public Health. 2024;17: 102510.

59. Scachetti GC, Forato J, Claro IM, Hua X, Salgado BB, Vieira A, et al. Reemergence of Oropouche virus between 2023 and 2024 in Brazil. medRxiv; 2024. p. 2024.07.27.24310296. doi:[10.1101/2024.07.27.24310296](https://doi.org/10.1101/2024.07.27.24310296)

60. Medlin S, Deardorff ER, Hanley CS, Vergneau-Grosset C, Siudak-Campfield A, Dallwig R, et al. Serosurvey of selected arboviral pathogens in free-rangins, two-toed sloths(Choloepus hoffmanni) and three-toed sloths (Bradypus variegatus) in Costa Rica, 2005-07. Journal of Wildlife Diseases. 2016;52: 883–892. doi:[10.7589/2015-02-040](https://doi.org/10.7589/2015-02-040)

61. Laroque PO, Valença-Montenegro MM, Ferreira DRA, Chiang JO, Cordeiro MT, Vasconcelos PFC, et al. Levantamento soroepidemiológico para arbovírus em macaco-prego-galego (Cebus flavius) de vida livre no estado da Paraíba e em macaco-prego (Cebus libidinosus) de cativeiro do nordeste do Brasil. Pesquisa Veterinária Brasileira. 2014;34: 462–468. doi:[10.1590/S0100-736X2014000500013](https://doi.org/10.1590/S0100-736X2014000500013)

62. Batista PM, Andreotti R, Chiang JO, Ferreira MS, Vasconcelos PFDC. Seroepidemiological monitoring in sentinel animals and vectors as part of arbovirus surveillance in the state of Mato Grosso do Sul, Brazil. Revista da Sociedade Brasileira de Medicina Tropical. 2012;45: 168–173. doi:[10.1590/S0037-86822012000200006](https://doi.org/10.1590/S0037-86822012000200006)

63. Pauvolid-Corrêa A, Campos Z, Soares R, Nogueira RMR, Komar N. Neutralizing antibodies for orthobunyaviruses in Pantanal, Brazil. PLOS Neglected Tropical Diseases. 2017;11: e0006014. doi:[10.1371/journal.pntd.0006014](https://doi.org/10.1371/journal.pntd.0006014)

64. Turell MJ, Gozalo AS, Guevara C, Schoeler GB, Carbajal F, López-Sifuentes VM, et al. Lack of Evidence of Sylvatic Transmission of Dengue Viruses in the Amazon Rainforest Near Iquitos, Peru. Vector-Borne and Zoonotic Diseases. 2019;19: 685–689. doi:[10.1089/vbz.2018.2408](https://doi.org/10.1089/vbz.2018.2408)

65. Batista PM, Andreotti R, de Almeida PS, Marques AC, Rodrigues SG, Chiang JO, et al. Detection of arboviruses of public health interest in free-living New World primates (*Sapajus* spp.; *Alouatta* *Caraya*) captured in Mato Grosso do Sul, Brazil. Revista da Sociedade Brasileira de Medicina Tropical. 2013-Nov-Dec;46: 684–690. doi:[10.1590/0037-8682-0181-2013](https://doi.org/10.1590/0037-8682-0181-2013)

66. Pereira-Silva JW, Ríos-Velásquez CM, de Lima GR, dos Santos EFM, Belchior HCM, Luz SLB, et al. Distribution and diversity of mosquitoes and Oropouche-like virus infection rates in an Amazonian rural settlement. PLOS ONE. 2021;16: e0246932. doi:[10.1371/journal.pone.0246932](https://doi.org/10.1371/journal.pone.0246932)

67. Tauro L, Cardoso C, Lima R, Nascimento L, dos Santos D, Campos GS, et al. An outbreak of Chikungunya: Tracking the epidemic threat in Salvador, Brazil. American Journal of Tropical Medicine and Hygiene. 2018;99: 53.

68. da Silva Ferreira R, de Toni Aquino da Cruz LC, de Souza VJ, da Silva Neves NA, de Souza VC, Filho LCF, et al. Insect-specific viruses and arboviruses in adult male culicids from Midwestern Brazil. Infection, Genetics and Evolution. 2020;85: 104561. doi:[10.1016/j.meegid.2020.104561](https://doi.org/10.1016/j.meegid.2020.104561)

69. Dias HG, Familiar-Macedo D, Garrido IO, dos Santos FB, Pauvolid-Corrêa A. Exposure of domestic animals to Mayaro and Oropouche viruses in urban and peri-urban areas of West-Central Brazil. One Health Outlook. 2024;6: 12. doi:[10.1186/s42522-024-00104-w](https://doi.org/10.1186/s42522-024-00104-w)

70. Feitoza LHM, de Carvalho LPC, da Silva LR, Meireles ACA, Rios FGF, Silva GS, et al. Influence of meteorological and seasonal parameters on the activity of *Culicoides* *Paraensis* (Diptera: Ceratopogonidae), an annoying anthropophilic biting midge and putative vector of Oropouche Virus in Rondônia, Brazilian Amazon. Acta Tropica. 2023;243: 106928. doi:[10.1016/j.actatropica.2023.106928](https://doi.org/10.1016/j.actatropica.2023.106928)

71. da Silva DMF, de Sousa FB, de Curcio JS, do Carmo Silva L, Annunciation CE, Salem-Izacc SM, et al. Transmissao vertical de arboviroses em Aedes aegypti em Goiania: Uma estrategia para a disseminacao de doencas transmitidas por mosquitos. Brazilian Journal of Infectious Diseases. 2023;27: 102812.

72. Bobrovitz, Niklas, et al. “SeroTracker‐RoB: A decision rule‐based algorithm for reproducible risk of bias assessment of seroprevalence studies.” *Research Synthesis Methods* 14.3 (2023): 414-426.
