# Supplementary figures and images for: Serological and viral prevalence of Oropouche virus (OROV): A systematic review and meta-analysis from 2000–2024 including human, animal, and vector surveillance studies
Source: PLoS Negl Trop Dis. 2025 Dec 29;19(12):e0013340. doi: 10.1371/journal.pntd.0013340 (PMC12799185; doi:10.1371/journal.pntd.0013340)

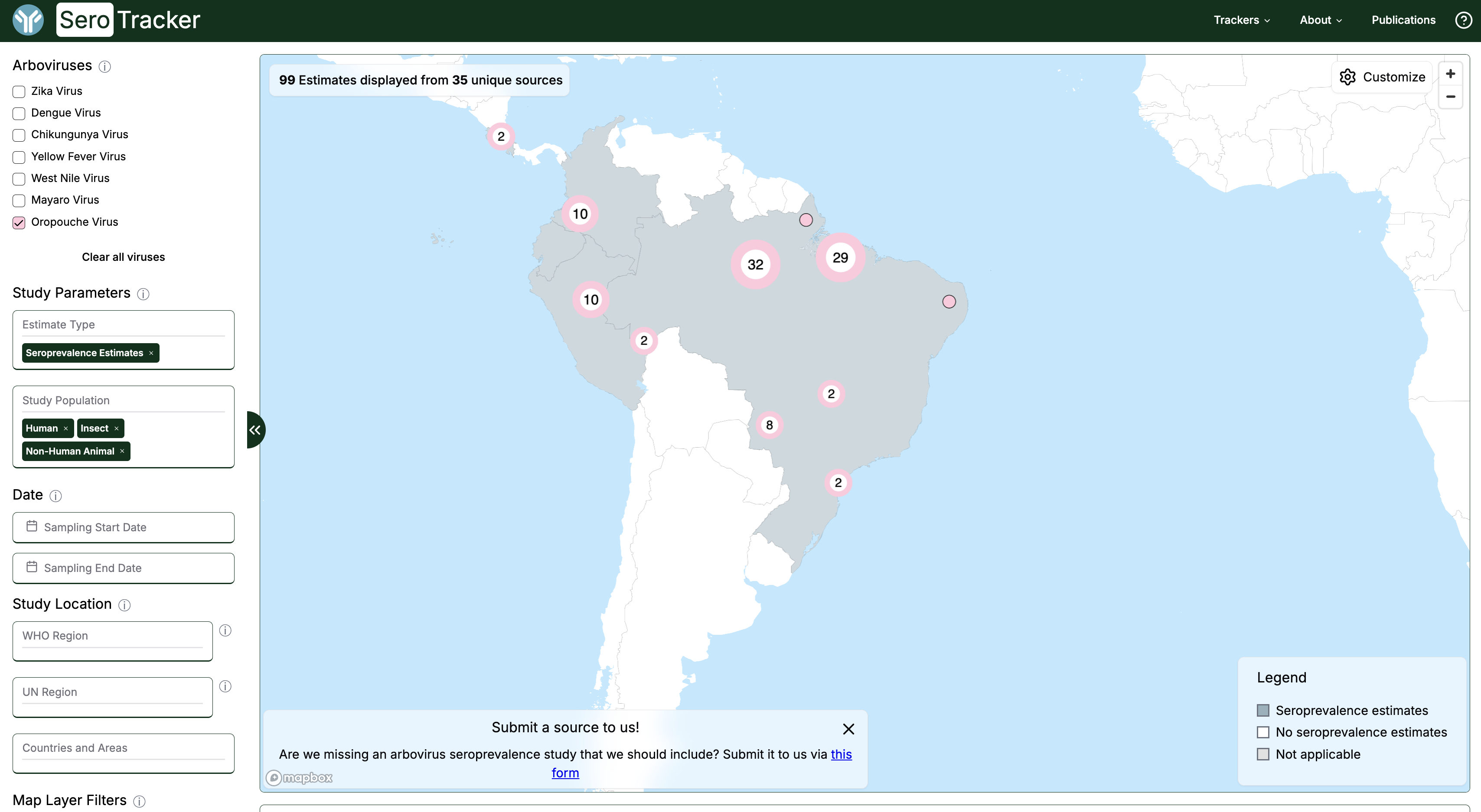

Supplement: S1 Fig — (PNG) [file pntd.0013340.s002.png]
