# Supplementary material for: Serological and viral prevalence of Oropouche virus (OROV): A systematic review and meta-analysis from 2000–2024 including human, animal, and vector surveillance studies
Source: PLoS Negl Trop Dis. 2025 Dec 29;19(12):e0013340. doi: 10.1371/journal.pntd.0013340 (PMC12799185; doi:10.1371/journal.pntd.0013340)
Supplement: Attachment — Submitted filename: OROV Paper Revisions 1_ Response to reviewers .docx [file pntd.0013340.s004.docx]

**2025-09 PLoS NTD Response to reviewers
Article title:** Serological and viral prevalence of Oropouche virus (OROV): A systematic review and meta-analysis from 2000-2024 including human, animal, and vector surveillance studies

**Original review feedback and comments are in black text. Author responses are in green.**

1) We ask that a manuscript source file is provided at Revision. Please upload your manuscript file as a .doc, .docx, .rtf or .tex. If you are providing a .tex file, please upload it under the item type u2018LaTeX Source Fileu2019 and leave your .pdf version as the item type u2018Manuscriptu2019.

**Response**: Thank you. We have uploaded the manuscript source file as directed.

2) Please upload all main figures as separate Figure files in .tif or .eps format. For more information about how to convert and format your figure files please see our guidelines:
<https://journals.plos.org/plosntds/s/figures>

**Response**: We have uploaded all figures as separate .tif files.

3) We have noticed that you have uploaded Supporting Information files, but you have not included a list of legends. Please add a full list of legends for your Supporting Information files after the references list.

**Response**: Thank you. We have added a list of legends for our supporting material after the reference section as suggested.

4) Thank you for stating "Study data will be made available through the interactive and open-access ArboTracker dashboard and data platform, accessible at <https://www.serotracker.com/pathogen/arbovirus/dashboard>." We strongly recommend all authors decide on a data sharing plan before acceptance, as the process can be lengthy and hold up publication timelines. Please note that, though access restrictions are acceptable now, your entire data will need to be made freely accessible if your manuscript is accepted for publication. This policy applies to all data except where public deposition would breach compliance with the protocol approved by your research ethics board.

**Response:** Our full dataset is now openly accessible on the ArboTracker dashboard, where we have added all Oropouche studies alongside the other seven arboviruses. We have interactive features to explore the qualifying literature and downloadable CSV files. This statement has been amended in the online submission form and in the manuscript draft.

5) Please ensure that the funders and grant numbers match between the Financial Disclosure field and the Funding Information tab in your submission form. Note that the funders must be provided in the same order in both places as well.

**Response**: We have modified as you suggested. Originally, we had listed some funders in the Financial Disclosure field for awards that were not relevant to this paper (with disclosures stating so) – we have now removed those for clarity. We also added any missing funders to the Funding Information section.

6) Please amend your Competing Interests statement in the online submission form. Please declare all competing interests beginning with the statement "I have read the journal's policy and the authors of this manuscript have the following competing interests:"

Note: If there are no competing interests to declare, please state "The authors have declared that no competing interests exist".

**Response:** We have amended the statement in the online submission form as requested.

6) As required by our policy on Data Availability, please ensure your manuscript or supplementary information includes the following:
- A numbered table of all studies identified in the literature search, including those that were excluded from the analyses.
- For every excluded study, the table should list the reason(s) for exclusion.

**Response:** This study was nested within a broader systematic review of arbovirus seroprevalence across seven other pathogens capturing publications from 1950 onwards, therefore we have not included this table due to the size of the search being in the many thousands, and the included/excluded articles not being necessarily representative of this paper’s analysis as it also includes Dengue, Zika, Yellow Fever, Mayaro, Chikungunya, and West Nile Virus information. We hope that the provided PRISMA flow diagram, search strategy, PROSPERO protocol, and full data dictionary/dataset at the open-access ArboTracker dashboard will be sufficient for reproducibility purposes.

- If any of the included studies are unpublished, include a link (URL) to the primary source or detailed information about how the content can be accessed.

**Response:** No unpublished studies were included. We did include preprints where they were published on an acceptable server and with a retrievable DOI, such as medRxiv.

- A table of all data extracted from the primary research sources for the systematic review and/or meta-analysis. The table must include the following information for each study:
 - Name of data extractors and date of data extraction
 - Confirmation that the study was eligible to be included in the review.
 - All data extracted from each study for the reported systematic review and/or meta-analysis that would be needed to replicate your analyses.

**Response:** This information is provided in Table A and B of our supplementary materials.

- If data or supporting information were obtained from another source (e.g. correspondence with the author of the original research article), please provide the source of data and dates on which the data/information were obtained by your research group.

**Response:** No data or supporting information was sourced outside our conducted search.

- If applicable for your analysis, a table showing the completed risk of bias and quality/certainty assessments for each study or outcome. Please ensure this is provided for each domain or parameter assessed. For example, if you used the Cochrane risk-of-bias tool for randomized trials, provide answers to each of the signalling questions for each study. If you used GRADE to assess certainty of evidence, provide judgements about each of the quality of evidence factor. This should be provided for each outcome.

**Response:** This information is available in Table C of our supplementary materials, with a full breakdown of each domain and parameter assessed in our modified Joanna Briggs Institute Tool in File E, also in the supplementary materials.

- An explanation of how missing data were handled.

**Response:** Generally, articles missing data in key variables were excluded at the screening stage based on our inclusion criteria, or excluded later. We have clarified this in lines 159-160.

This information can be included in the main text, supplementary information, or relevant data repository.

Note: If the reviewer comments include a recommendation to cite specific previously published works, please review and evaluate these publications to determine whether they are relevant and should be cited. There is no requirement to cite these works unless the editor has indicated otherwise.


**Reviewers' Comments:**

Reviewer's Responses to Questions

**Key Review Criteria Required for Acceptance?**

As you describe the new analyses required for acceptance, please consider the following:

**Methods**

-Are the objectives of the study clearly articulated with a clear testable hypothesis stated?

-Is the study design appropriate to address the stated objectives?

-Is the population clearly described and appropriate for the hypothesis being tested?

-Is the sample size sufficient to ensure adequate power to address the hypothesis being tested?

-Were correct statistical analysis used to support conclusions?

-Are there concerns about ethical or regulatory requirements being met?

Reviewer #1: The methods section should be described in greater detail. For instance, what type of generalized linear mixed-effects models were used? What distribution and link function were applied? This information is particularly relevant and is currently not adequately reported.
The authors should make a greater effort to identify and understand the sources of heterogeneity among the studies so that appropriate conclusions can be drawn from the results. A meta-regression analysis may be appropriate in this regard.

**Response:** We have added detail on the model to the methods section (lines 186-187). We have also added a meta-regression analysis (Table 3) to better identify and understand the sources of heterogeneity among the studies.

Reviewer #2: This is not a hypothesis-driven study
I am not concerned about the ethical or regulatory requirements being met.

**Response:** Thank you for your approval. No action items have been outlined so we will move on to the next comment.

**Results**

-Does the analysis presented match the analysis plan?

-Are the results clearly and completely presented?

-Are the figures (Tables, Images) of sufficient quality for clarity?

Reviewer #1: The analysis does match the analysis plan.
It is important to specify the within-country locations of the included studies. Generalizing results at the country level may overstate the findings. For example, reporting seroprevalence of 24.4% for Brazil or 14.5% for Colombia may be misleading. The studies reporting the highest seroprevalence in Brazil for instance (Azevedo et al., 2004; Vasconcelos et al., 2006) were conducted in the state of Pará, which accounts for only about 4% of the Brazilian population. Greater effort should be made to account for the within-country origin of the studies and, more importantly, the local characteristics of those areas.
Was any additional analysis performed to investigate the high heterogeneity observed in the results? Based on those values, can the authors reasonably justify grouping these studies together? Probably authors should explore alternative grouping of the results

**Response:** We have added the within-country locations of the included studies to forest plots (Figure 2-4). Due to the high heterogeneity, we have also modified the in-text reporting to describe the range of seroprevalence in each country, rather than the pooled estimate. We continue to group studies by country visually as it is the most relevant grouping for exploring the spread of Oropouche virus. Also note that the seroprevalence of 24.4% or 14.5% in Figure 2 are for febrile patients or suspected cases - we are not generalizing these results to the entire Brazilian or Colombian population. The heterogeneity is lower among general population studies (Figure 3).

Reviewer #2: Yes

**Response:** Thank you for your approval. No action items have been outlined so we will move on to the next comment.

**Conclusions**

-Are the conclusions supported by the data presented?

-Are the limitations of analysis clearly described?

-Do the authors discuss how these data can be helpful to advance our understanding of the topic under study?

-Is public health relevance addressed?

Reviewer #1: The conclusions should be refined to reflect a more accurate presentation of the results. It does not seem reasonable to use highly localized studies to draw country-wide conclusions.
Only by considering further refinement of results I would confidently state that conclusions are important for the understanding of the topic

**Response:** Thank you for your comment. We have refined our phrasing of country-specific results to ensure thoughtful representation of this issue throughout the paper where needed, with changes made to lines 278-283; 232-242; 253; and 211-215. In addition we have added a line in our limitations describing the challenge of synthesizing localized studies for broader population insights.

Reviewer #2: Yes

**Response:** Thank you for your approval. No action items have been outlined so we will move on to the next comment.

**Editorial and Data Presentation Modifications?**

Use this section for editorial suggestions as well as relatively minor modifications of existing data that would enhance clarity. If the only modifications needed are minor and/or editorial, you may wish to recommend “Minor Revision” or “Accept”.

Reviewer #1: Figures: Figure legends should be self-explanatory. For example, clarify what the numbers in brackets represent.

I suggest to authors improve literature support of the statements, specially in the introduction. For example,
First paragraph of introduction: “In addition, early data raise the concern that OROV’s vector range may be expanding both geographically and into other carrying vectors beyond the biting midge, for example Culex mosquitoes” This sentence needs a reference.
Second paragraph of introduction: “Additionally, evidence of infection in animal reservoirs like birds and mammals remains poorly defined, complicating efforts to assess zoonotic spillover risks”. Needs a reference.

**Response:** Thank you for your suggestion. We have modified figure legends to be self-explanatory. We have also reviewed the introduction to ensure adequate supporting evidence, and added citations to the two quotes in particular that you have highlighted. See: <https://pubmed.ncbi.nlm.nih.gov/40284935/> and <https://onehealthoutlook.biomedcentral.com/articles/10.1186/s42522-024-00104-w>

Reviewer #2: (No Response)

**Response:** No response has been outlined so we will move on to the next comment.

**Summary and General Comments**

Use this section to provide overall comments, discuss strengths/weaknesses of the study, novelty, significance, general execution and scholarship. You may also include additional comments for the author, including concerns about dual publication, research ethics, or publication ethics. If requesting major revision, please articulate the new experiments that are needed.

Reviewer #1: This is a relevant study. However, a major concern is the broad generalization of the results. Grouping studies solely by country without considering their local context can be misleading. For instance, stating that febrile individuals in Brazil have an Oropouche seroprevalence of 24.4% is problematic. Closer examination reveals that most of the studies reporting the highest values were conducted in municipalities located in highly sylvatic areas of Brazil (e.g., Amazonas and Pará). Such values should be attributed to those specific regions rather than the entire country, as the current approach may significantly distort the interpretation of the results.

Additionally, the reported heterogeneity is extremely high—above 95%. This issue does not appear to be adequately addressed, especially given the presence of very low estimates (e.g., prevalence between 0.2% and 3.5%). Further analyses are warranted to explain and understand the sources of variability. Possible approaches include meta-regression or more detailed intra-group analyses. It is also possible that important local variables, not considered in the current analysis, could explain part of this variability.

**Response:** Thank you for your overall comments. We have addressed the concerns regarding grouping studies by country and high heterogeneity below under Major Comments.

Reviewer #2: This manuscript presents a systematic review and meta-analysis of OROV serological and viral prevalence across human, animal, and vector populations from 2000-2024. The authors identify 71 articles and provide pooled prevalence estimates for humans, as well as a descriptive synthesis for animal and vector data. The review is well-motivated, the methods are largely appropriate, and the open-access data dashboard (ArboTracker) is a valuable resource. The manuscript would benefit from clarifications in data presentation, additional detail in the methods, and a more explicit discussion of certain limitations.

**Response:** Thank you for your positive comments on our manuscript and thoughtful suggestions. We have addressed your comments by clarifying our data, methods, and limitations, with more detail on our changes in the specific comments below.

Major Comments

1. The manuscript states that 71 articles were included, but only 61 (human) and 96 (vector) studies were sampled after 2000 for meta-analysis. The text should explicitly state which data were meta-analyzed vs. only described, and why the 2000 cutoff was chosen. Please also clarify how multiple estimates from the same source were handled, e.g., were they pooled within-study or treated as separate entries?

**Response:** We mention in lines 183-185 that the 2000 cutoff was chosen to “capture timely evidence from the last decade and provide a current-day snapshot of the virus’s circulation.” On multiple estimates, we have clarified in lines 160-163, “Where an article or source contains multiple prevalence estimates stratified by time, geography, non-overlapping populations, test type, or gender, we split the article into multiple “studies”—for the purpose of this review, “study” means a distinct estimate *treated as separate entries for analysis*.”

2. The I2 values for the pooled estimates are extremely high (≥ 92-99%). While heterogeneity is acknowledged, the manuscript would benefit from more formal exploration, e.g., meta-regression or sensitivity analysis by test type, sampling frame, or outbreak vs. non-outbreak context.

**Response:** We have added a sensitivity analysis by test type to the Supplement. We already present results stratified by sampling frame in the main text (febrile illness or suspected cases vs general asymptomatic populations). We have also added a meta-regression (Table 3) to better identify and understand the sources of heterogeneity among the studies.

3. As diagnostic assay performance is a major confounder (particularly cross-reactivity in ELISA/HAI), stratifying or adjusting by assay type could improve interpretability.

**Response:** We ran an analysis stratified by assay performance; however, we found that population type was the biggest confounder so that is what we stratified by in the main text. We have added a sensitivity analysis of pooled prevalence by assay type to the Supplement, and a meta-regression (Table 3) with assay type as a covariate.

4. The limitations section mentions cross-reactivity and poor reporting of assay details, but the impact on pooled prevalence is not quantified. Could you provide a summary table indicating which studies used confirmatory neutralization tests vs. screening assays only? This would allow readers to assess confidence in different subsets of the data.

**Response:** We have added details on which studies used confirmatory neutralization tests vs. screening assays only to Table 1. We have also added a sensitivity analysis of pooled prevalence by assay type to the Supplement, and a meta-regression (Table 3) with assay type as a covariate, showing that seroprevalence estimates were comparable.

5. The descriptive synthesis of animal and vector prevalence is valuable but currently feels secondary. The text could be strengthened by summarizing the geographic overlap between human and vector detection. Commenting on the time gap in vector studies, e.g., no studies after 2018 in certain groups, and discussing vector competence evidence in more detail, referencing the limited number of studies showing OROV RNA in non-traditional vectors.

**Response:** Thank you for the suggestion. We have added a section commenting on these points and summarizing the geographic overlap between human and vector detection in lines 376-385.

6. The automated decision-rule approach is innovative, but for transparency, the manuscript should provide a brief explanation of the six automated items vs. two manually reviewed items. An example of how different combinations map to low/moderate/high ratings. Clarify if human, vector, and animal studies were assessed using the exact same criteria.

**Response:** Thank you for this suggestion. We have added a reference to Supplementary File E, where we provide an explanation of the six automated items vs. two manually reviewed items, with examples. We have also clarified that human and vector studies were assessed using the same criteria.

7. Figure 2 and Figure 3: Consider adding the number of studies (n) and total sample size directly in the plot captions or alongside the pooled estimates.
8. Figure 5: Label n and sample size. For all forest plots, displaying raw proportions alongside CIs would make them easier to interpret.
9. Some forest plots (esp. Figure 2) are difficult to read due to density; splitting by country or using facet plots could help.

**Response to 7-9:** Thank you for the suggestions on the figures - we have added labels for sample size and number of positives to aid interpretation.

Minor Comments

1. Introduction “…OROV may additionally be transmitted sexually and…” I may reword this because it is too early to speculate.

**Response:** We have implemented suggesting wording changes.

2. Line 172: "i.e., with fever and related symptoms" replace “i.e.” with “that is” or rephrase for smoother readability.

**Response:** We have implemented suggesting wording changes.

3. Ensure consistent terminology: the manuscript alternates between “non-human animals” and “animal hosts”. Pick one and define early.

**Response:** We have decided on ‘animal hosts’ for our consistent terminology and have changed instances throughout. We also defined it in line 130.

4. Some tables (e.g., Table 2) contain a lot of detail; consider moving full species lists to supplementary materials for readability.

**Response:** We agree and have moved the species list in Table 2 to the supplement, but have otherwise kept the rest of the table in the main text.

5. The Discussion could explicitly mention that prevalence estimates in humans may be biased upward in febrile cohorts due to convenience sampling.

**Response**: We have added a statement on this into the discussion of our limitations in lines 410-411.

6. The conclusion mentions “increasing severity” of OROV epidemics; severity metrics are not presented in this paper, so this should be reworded unless referencing external data.

**Response:** We have removed reference to “increasing severity.”
